# Optimizing Interfacial Charge Dynamics and Quantum Effects in Heterodimensional Superlattices for Efficient Hydrogen Production

**DOI:** 10.1002/advs.202412805

**Published:** 2024-12-16

**Authors:** Jinpeng Li, Weikang Dong, Zibo Zhu, Yang Yang, Jiadong Zhou, Sufan Wang, Yao Zhou, Erhong Song, Jianjun Liu

**Affiliations:** ^1^ State Key Lab of High‐Performance Ceramics and Superfine microstructure Shanghai Institute of Ceramics Chinese Academy of Sciences Shanghai 200050 China; ^2^ School of Physics Beijing Institute of Technology Beijing 100081 China; ^3^ Center of Materials Science and Optoelectronics Engineering University of Chinese Academy of Sciences Beijing 100049 China; ^4^ Advanced Research Institute of Multidisciplinary Science Beijing Institute of Technology Beijing 100081 China; ^5^ College of Chemistry and Materials Science Anhui Normal University Wuhu 241000 China; ^6^ School of Chemistry and Materials Science, Hangzhou Institute for Advanced Study University of Chinese Academy of Science 1 Sub‐lane Xiangshan Hangzhou 310024 China

**Keywords:** charge separation, heterodimensional superlattice, hydrogen evolution, quantum localization

## Abstract

Superlattice materials have emerged as promising candidates for water electrocatalysis due to their tunable crystal structures, electronic properties, and potential for interface engineering. However, the catalytic activity of transition metal‐based superlattice materials for the hydrogen evolution reaction (HER) is often constrained by their intrinsic electronic band structures, which can limit charge carrier mobility and active site availability. Herein, a highly efficient electrocatalyst based on a VS_2_‐VS heterodimensional (2D‐1D) superlattice with sulfur vacancies is designed addressing the limitations posed by the intrinsic electronic structure. The enhanced catalytic performance of the VS_2_‐VS superlattice is primarily attributed to the engineered heterojunction, where the work function difference between the VS_2_ layer and VS chain induces a charge separation field that promotes efficient electron‐hole separation. Introducing sulfur vacancies further amplifies this effect by inducing quantum localization of the separated electrons, thereby significantly boosting HER activity. Both theoretical and experimental results demonstrate that the superlattice achieves a ΔG_H*_ of −0.06 eV and an impressively low overpotential of 46 mV at 10 mA·cm^−2^ in acidic media, surpassing the performance of commercial Pt/C while maintaining exceptional stability over 15 000 cycles. This work underscores the pivotal role of advanced material engineering in designing catalysts for sustainable energy applications.

## Graphical abstract

In this study, the VS_2_‐VS heterodimensional superlattice electrocatalyst with sulfur vacancies is designed to overcome the intrinsic electronic constraints. The engineered heterojunction between the VS_2_ layer and the VS chain creates a charge separation field that enhances electron‐hole separation, while sulfur vacancies contribute to quantum localization, significantly boosting HER activity.

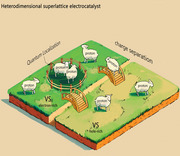

## 1 Introduction

Electrocatalytic water splitting (2H_2_O → O_2_ + 2H_2_) is widely regarded as a green and efficient way for hydrogen production, offering a promising solution to the energy crisis and environmental issues caused by reliance on traditional fossil fuels.^[^ ^1^, ^2^, ^3^ ^]^ While commercial Pt/C and IrO_2_/RuO_2_ are excellent water‐splitting catalysts, their scarcity limits widespread use, promoting research into non‐noble metal alternatives. Although these alternatives show promise at low current densities, they still struggle with high cell voltages (1.8–2.4 V), significantly above the thermodynamic potential (1.23 V), due to suboptimal electrocatalytic activity.^[^ ^4^, ^5^, ^6^, ^7^ ^]^ To efficiently drive catalytic processes at low overpotentials with sufficiently fast kinetics, significant efforts in recent years have focused on developing electrocatalysts to enhance performance and identify ideal substitutes for noble metal‐based catalysts.^[^ ^8^, ^9^, ^10^, ^11^, ^12^, ^13^ ^]^ Currently, the commonly used strategies to improve the electrocatalytic performance are defects engineering,^[^ ^14^, ^15^, ^16^, ^17^, ^18^ ^]^ heteroatom doping,^[^ ^19^, ^20^, ^21^, ^22^, ^23^, ^24^, ^25^ ^]^ crystal phase regulation,^[^ ^26^, ^27^, ^28^, ^29^ ^]^ conductive carbon materials as support,^[^ ^30^, ^31^, ^32^, ^33^ ^]^ heterostructure/interface engineering,^[^ ^34^, ^35^, ^36^, ^37^, ^38^ ^]^ etc. Drawing upon earlier pioneering research and our previous studies, enhancing catalyst performance primarily involves modifying the intrinsic electronic structure of catalysts. This modification essentially pertains to charge separation and quantum localization, which are critical for determining the catalytic activity of electrocatalysts.^[^ ^39^, ^40^, ^41^, ^42^, ^43^ ^]^ Achieving and controlling efficient charge separation and targeted localization is key to significantly enhancing the catalytic activity for hydrogen production in water electrolysis.

Superlattice materials, composed of alternating monolayer or few‐layer nanosheets (A and B) in a precise stacking sequence, have emerged as a promising solution for achieving efficient charge separation and quantum localization, essential for enhancing catalytic performance.^[^ ^44^, ^45^, ^46^, ^47^, ^48^ ^]^ Charge separation, which involves effectively separating electron‐hole pairs across different layers, and quantum localization, where electrons are confined to specific regions, thereby increasing the density of active sites, are crucial for enhancing catalytic performance.^[^ ^49^, ^50^, ^51^, ^52^, ^53^ ^]^ Unlike randomly stacked nanocomposites, superlattices are characterized by their strictly ordered stacking sequences, which inherently promote efficient charge separation and quantum localization. These processes, in turn, give rise to unique physicochemical properties, including ultrafast interlayer charge transfer and a high density of active sites, both of which are critical for enhancing catalytic performance.^[^ ^54^, ^55^ ^]^ Catalytic reactions within superlattice materials can be precisely engineered on specific chemically active surfaces, sites, or interfaces by carefully selecting coupling layers that optimize intermediate adsorption behaviors.^[^ ^56^, ^57^, ^58^ ^]^ The ability of superlattices to inherently facilitate efficient charge separation and quantum localization further enhances these interactions, making them highly effective in driving catalytic processes. As a result, superlattice materials are exceptionally promising candidates for advanced applications in water electrolysis, where enhanced catalytic efficiency is crucial.^[^ ^59^ ^]^ Similarly, among various non‐precious metal materials, vanadium sulfides have shown remarkable performance in water splitting, owing to targeted structural regulation, and have been extensively studied.^[^ ^60^, ^61^, ^62^, ^63^, ^64^ ^]^ Recently, the anomalous Hall effect was observed at room temperature in heterodimensional superlattice structures with vanadium sulfide, highlighting the unique electronic properties of these materials.^[^ ^48^ ^]^ The heterodimensional structure, characterized by its spatially distinct regions with varied electronic environments, is particularly advantageous for achieving efficient charge separation and quantum localization. This configuration enhances the distribution and mobility of charge carriers, which are crucial for driving catalytic reactions. As a result, these heterodimensional superlattices hold significant potential for improving catalytic performance in water electrolysis, making them promising candidates for such applications.

Herein, we design a highly efficient electrocatalyst for hydrogen evolution reaction (HER) based on a VS_2_‐VS (2D‐1D) heterodimensional superlattice with strategically introduced sulfur vacancies. This innovative superlattice design overcomes the limitations of transition metal‐based superlattices by creating a distinct heterojunction. The work function difference between the VS_2_ layer and VS chain generates a charge separation field, which enhances electron‐hole separation and promotes efficient HER activity. Additionally, the introduction of sulfur vacancies optimizes hydrogen adsorption by inducing quantum localization of electrons, leading to a significant improvement in HER performance. Both theoretical calculations and experimental measurements reveal that the superlattice exhibits a ΔG_H*_ of −0.06 eV and a remarkably low overpotential of 46 mV at a current density of 10 mA·cm^−2^ in acidic conditions. This performance not only exceeds that of commercial Pt/C catalysts but also demonstrates outstanding stability over 15 000 cycles. This work highlights the effectiveness of designing superlattice materials with tailored structural features, providing a promising strategy for developing high‐performance and stable electrocatalysts for sustainable hydrogen production.

## 2 Results and Discussion

**Figure 1:**
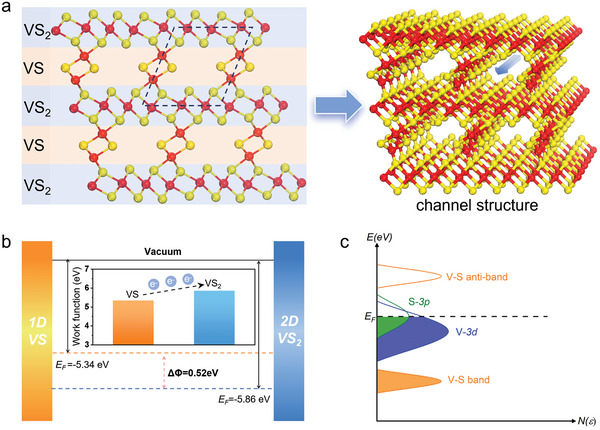
Diagram of VS_2_‐VS heterodimensional superlattice structure and strategies for charge separation and quantum localization. a) 2D and 3D Diagram of VS_2_‐VS superlattice model. The black dotted border indicates the channel structure inside the VS_2_‐VS superlattice. The arrow indicates the extension direction of the 1D VS chain. b) Schematic diagram of charge transfer based on the work function of 1D VS chain and 2D VS_2_ layer. c) The charge regulation effect on manipulating active electronic states in V_S_‐VS_2_‐VS superlattice based on defect engineering.

As shown in **Figure** **1**a, the heterodimensional superlattice structure consists of alternating VS_2_ layers and VS chains, arranged in a periodic structure. In contrast to traditional 2D‐2D superlattices, the VS_2_‐VS superlattice forms a unique channel structure at the interfaces between the 2D and 1D structures, thereby exposing sulfur atoms on VS and VS_2_ components. The porous structure provides highly exposed active sites that act as effective proton adsorption points, facilitating the kinetics of hydrogen adsorption and desorption, thereby significantly enhancing the HER process.

In Figure 1b, the difference in work functions between the VS_2_ layers (5.86 eV) and VS chains (5.34 eV), as determined from calculations, induces separation of electrons and holes, thereby generating an intrinsic charge separation field that facilitates their efficient segregation. Bader charge calculations before and after forming the heterodimensional superlattice reveal an increase in electrons on the VS_2_ layer and a decrease in the VS chains (Figure S1, Supporting Information), confirming effective electron‐hole separation. This redistribution makes the electron‐rich VS_2_ layer more favorable for proton adsorption, thereby optimizing the H* adsorption energy for HER.

Following the initial enhancement of catalytic activity achieved through effective charge separation, the introduction of quantum localization further intensifies these effects. This advanced phenomenon ensures that the spatial confinement of charge carriers within specific orbitals leads to significantly improved catalytic efficiency. As illustrated in Figure 1c, sulfur vacancies induce quantum localization of the separated electrons, causing their accumulation near the Fermi level, primarily within the V‐3*d* and S‐3*p* orbitals. This quantum localization optimizes hydrogen adsorption, thereby significantly boosting HER activity by fine‐tuning the H* adsorption energy.

To study the surface catalytic activity of the material, slab models with VS_2_ (001) and VS (001) surfaces were constructed and named 3VS_2_‐2VS and 3VS_2_‐3VS according to the differences in multilayer structures (see Figures S2 and S3, Supporting Information). The Gibbs free energy of hydrogen adsorption (ΔG_H*_) is a crucial metric for linking theoretical predictions to experimental observations of catalytic activity. An optimal ΔG_H*_ value of 0.0 eV represents a thermoneutral state, enabling the adsorbed hydrogen atom to effectively participate in proton/electron‐coupled reactions and facilitate the release of molecular hydrogen. The ΔG_H*_ value for the VS_2_ (001) surface is 0.50 eV, indicating that the VS_2_ surface is essentially inert to HER. In contrast, the VS (001) surface, which exposes V atoms as active adsorption sites, demonstrates a significantly stronger hydrogen adsorption capability, with a ΔG_H*_ of −0.24 eV. However, the HER site density on the material surface is much lower than that of the S sites on the channel structures, which are abundant within the superlattice. This is attributed to the unique advantages of the heterodimensional structure of the VS_2_‐VS. Therefore, we focus on the channels within the superlattice as the primary region for investigating HER activity.

**Figure 2:**
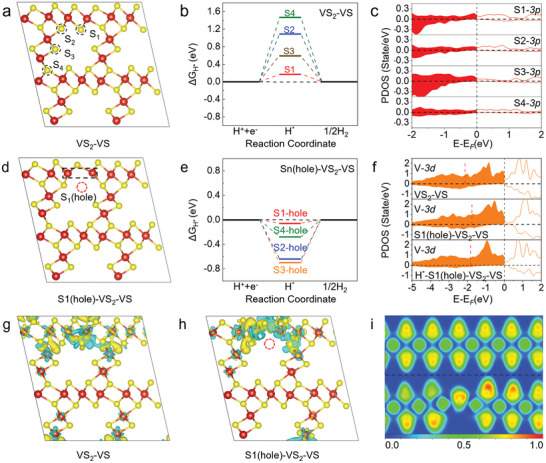
a) VS_2_‐VS models with four possible absorption sites, labeled S1, S2, S3, and S4, respectively. b) The calculated ΔG_H*_ of the four possible adsorption sites of VS_2_‐VS in HER. c) The PDOS of four active S sites of VS_2_‐VS. d) S1(hole)‐VS_2_‐VS models used to describe defect structure. (red circles indicate sulfur defects) e) The calculated ΔG_H*_ of four defect structures in HER. f) The PDOS of vanadium atoms (black rectangles of the panel d) of VS_2_‐VS, S1(hole)‐VS_2_‐VS and H*‐S1(hole)‐VS_2_‐VS. The position of the d‐band center of V‐*3d* is indicated by the red dashed line. g,h) Charge density difference plots of VS_2_‐VS and S1(hole)‐VS_2_‐VS, with yellow and blue regions indicating electron accumulation and depletion around the interface, respectively. i) Contour line diagrams of the electron localization function (ELF) for VS_2_‐VS and S1(hole)‐VS_2_‐VS. Labeled as the color scale of the values. (warmer colour region indicates electrons are more localised within this region).

Based on the channel structure of the VS_2_‐VS superlattice, four potential active sites for hydrogen adsorption have been identified and labeled as S1, S2, S3, and S4 (**Figure** **2**a; Figure S4, Supporting Information). These sites are predominantly situated at the interface between the VS_2_ layer and the VS chain, where the sulfur atoms coordinate with the surrounding vanadium atoms. As shown in Figure 2b, theoretical analysis indicates that S1 is the most active site, with a ΔG_H*_ of 0.17 eV, which is significantly lower than those of the S2–S4 sites. This unique coordination environment is critical for catalytic activity, as the interface between the VS_2_ layer and the VS chain promotes efficient charge and hole separation, leading to a higher concentration of electrons at the S1 site, thereby enhancing hydrogen adsorption.^[^ ^65^, ^66^ ^]^ Compared to the ΔG_H*_ of pure VS_2_ (0.35 eV) and VS (−0.40 eV), the VS_2_‐VS with a heterojunction interface exhibits superior HER activity (Figure S5, Supporting Information). This provides direct evidence that the charge separation at the VS_2_‐VS interface offers an optimal balance for hydrogen adsorption. Further analysis of the projected density of states (PDOS) at different sites (Figure 2c) reveals that the 3*p* orbitals of S1 exhibit a higher density of electronic states near the Fermi level (−0.5 to 0 eV) compared to the S2–S4 sites. The increased density of states at S1 contributes to more favorable adsorption energy and supports efficient charge separation, further enhancing its catalytic activity. To further enhance catalytic performance, we introduced sulfur vacancies into the internal channel structure of the VS_2_‐VS superlattice. This modification not only tuned the electronic structure but also induced quantum localization effects, effectively confining charge carriers to specific regions and further enhancing the catalyst's overall efficiency.

The strategic incorporation of sulfur vacancies (S‐vacancies) serves as a potent approach to amplify catalytic properties by harnessing the effects of quantum localization. As shown in Figure 2d and Figure S6 (Supporting Information), we systematically introduced S‐vacancies at four distinct sites within the VS_2_‐VS superlattice (V_S_‐VS_2_‐VS), resulting in the defect structures S1(hole)‐VS_2_‐VS, S2(hole)‐VS_2_‐VS, S3(hole)‐VS_2_‐VS, and S4(hole)‐VS_2_‐VS, each with a defect concentration of 3.125%. These vacancies provided an opportunity to explore the role of quantum localization in boosting hydrogen adsorption. To evaluate this, we performed calculations for each S‐vacancy system, focusing on the hydrogen adsorption free energy at the possible adsorption sites (Figures S7–S11, Supporting Information). Our results identified the S1 vacancy as the most favorable site, with an optimal ΔG_H*_ of −0.06 eV (Figure 2e). This finding demonstrates that the quantum localization induced by the S‐vacancy significantly enhances catalytic activity by confining charge carriers in localized regions around the defect, thereby strengthening the interaction between the catalyst and hydrogen atoms. Building on the above research, we further analyzed the electronic density of states in different vacancy systems, focusing primarily on the V‐3*d* and S‐3*p* electronic states near the Fermi level (Figures S12–S16, Supporting Information). Further analysis of the PDOS revealed that the formation of the S1 vacancy significantly increases the V‐*3d* states near the Fermi level (−1 to 0 eV), thereby facilitating stronger hydrogen adsorption at the S1 site (Figure 2f). As shown in Table S1 (Supporting Information), the formation of S vacancies led to a shift in the d‐band center of the V‐*3d* toward the Fermi level, from −2.13 to −1.76 eV. Following hydrogen adsorption, some electrons from the V atom are transferred to the hydrogen proton, decreasing the electronic states near the Fermi level, which results in a shift of the d‐band center from −1.76 to −1.83 eV. This further confirms that the electronic states near the Fermi level play a crucial role in hydrogen adsorption. These findings underscore how defect engineering, driven by quantum localization, effectively optimizes the electronic structure and enhances the HER activity of the catalyst.

To more intuitively investigate the electronic structure and confirm the charge separation and quantum localization effects in the system, we analyzed the charge density difference plots for both VS_2_‐VS and S1(hole)‐VS_2_‐VS, as shown in Figure 2g,h. Compared to the S1 site on VS_2_‐VS, a pronounced electron‐gaining region is observed near the S‐vacancy, indicating significant electron localization caused by charge redistribution in the defect structure. This localization enhances the adsorption capacity of the sulfur vacancy, effectively lowering the hydrogen adsorption energy barrier. Additionally, the electron localization function (ELF) maps comparing the perfect superlattice with the S‐defect‐containing VS_2_‐VS reveal that atoms near the S‐vacancy exhibit higher charge densities (see Figure 2i). To further demonstrate the impact of defect engineering on the electronic properties of materials, particularly the enhancement of electron localization, Bader charge calculations for VS_2_‐VS and S1(hole)‐VS_2_‐VS are provided in Table S2 (Supporting Information). This analysis quantitatively shows that, compared to the V atoms in the VS_2_‐VS, the sulfur vacancies (S1(hole)‐VS_2_‐VS) exhibit a higher charge distribution. Specifically, the V atoms near the defect accumulate more charge, with the Bader charges for V1 and V8 being 11.72 and 11.82, respectively, compared to 11.57 and 11.59 for V1 and V8 in the undisturbed VS_2_‐VS configuration. This observation is consistent with our previous electronic structure analysis of the defective systems, further supporting the role of quantum localization. In the hydrogen adsorption structure (see Figure S17, Supporting Information), it is observed that when hydrogen is adsorbed at the sulfur vacancy site on the VS_2_‐VS superlattice, electrons accumulate around the hydrogen atom, enhancing its adsorption. Finally, to more accurately analyze the electronic structure of the material, the band structure of S1(hole)‐VS_2_‐VS was calculated using the Heyd‐Scuseria‐Ernzerhof (HSE06) exchange‐correlation functional. This function is widely regarded as reliable for evaluating band gaps. As shown in Figure S18 (Supporting Information), S1(hole)‐VS_2_‐VS exhibits half‐metallic property, with the valence band and conduction band of the spin‐up bands overlapping, and the spin‐down bands having a band gap of only 0.39 eV. Therefore, the material possesses great conductivity, and electrons can transfer rapidly within the systems, which is crucial for enhancing the performance of electrocatalysts in processes such as HER.

**Figure 3:**
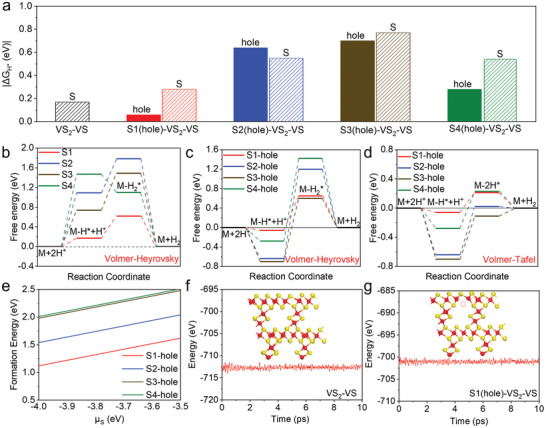
a) The computed lowest ΔG_H*_ for different models. b) Free energy diagram for the HER following the Volmer–Tafel pathway on the VS_2_‐VS model. c,d) Free energy diagram for the HER following the Volmer–Heyrovsky pathway (c) and the Volmer–Tafel pathway (d) on various defect models. e) The defect forming energy of four defect models. f,g) The energy fluctuation with time step at 300 K of VS_2_‐VS and S1(hole)‐VS_2_‐VS from AIMD calculation.

Understanding the dominant pathway is crucial for optimizing catalytic performance and designing efficient catalysts for hydrogen production. In **Figure** **3**, the kinetics of the hydrogen evolution reaction (HER) on the VS_2_‐VS superlattice, both pristine and with sulfur vacancies, are analyzed to determine whether the reaction follows the Volmer–Heyrovsky or Volmer–Tafel mechanism. Adsorption energies for the optimal sites across all configurations, including the pristine VS_2_‐VS and the four defect structures, were evaluated, as shown in Figure 3a. Among these, the S1(hole)‐VS_2_‐VS configuration was found to have a |ΔG_H*_| value closest to the ideal for hydrogen evolution, suggesting it is a particularly favorable site for further investigation.

In Figure 3b, for the VS_2_‐VS configuration, the analysis was then extended to the four identified sulfur adsorption sites (S1–S4), where the reaction mechanisms were explored in more detail. The simulated pathways for hydrogen evolution at these sites revealed that the Heyrovsky step is the rate‐determining step (RDS) at the S1 site, with an energy barrier of 0.75 eV. This barrier is lower than those at the other three sites, indicating that S1 is the most favorable site for HER. This finding is consistent with earlier results, where S1 was identified as the most active site based on the adsorption of a single hydrogen atom.

Free energy diagrams for both the Volmer–Heyrovsky and Volmer–Tafel pathways were constructed to reveal the HER mechanisms in the presence of S‐vacancies (Figure 3c,d). The Gibbs free energy barriers for this step across the various defect sites were calculated as 0.71 eV for S1(hole)‐VS_2_‐VS, 1.84 eV for S2(hole)‐VS_2_‐VS, 1.30 eV for S3(hole)‐VS_2_‐VS, and 1.30 eV for S4(hole)‐VS_2_‐VS. For the Volmer–Heyrovsky pathway, the Heyrovsky step was identified as the rate‐determining step (RDS) at the equilibrium potential. These values indicate significant energy barriers that impede the HER process along this pathway. In contrast, for the Volmer–Tafel pathway, the energy barrier for the Tafel step was consistently lower than for the Volmer step across all defect models, resulting in overall Gibbs free energy changes that were smaller than those of the Volmer–Heyrovsky pathway. Specifically, the Tafel step energy barriers were 0.27 eV for S1(hole)‐VS_2_‐VS, 0.66 eV for S2(hole)‐VS_2_‐VS, 0.59 eV for S3(hole)‐VS_2_‐VS, and 0.51 eV for S4(hole)‐VS_2_‐VS. This lower energy barrier suggests that the Volmer–Tafel pathway is more energetically favorable for HER. Thus, the energy barrier comparison suggests that HER on the VS_2_‐VS superlattice, particularly with sulfur vacancies, predominantly follows the Volmer–Tafel pathway, with the Tafel step as the rate‐determining step. The S1‐vacancy stands out as the most favorable site, offering the lowest energy barrier and optimal conditions for HER.

To evaluate the stability of the four defect configurations, thermodynamic and kinetic calculations were performed. Figure 3e shows a linear correlation between the sulfur chemical potential and the defect formation energy of S‐vacancies, with values of −4.12 eV under S‐rich conditions and −3.46 eV under V‐rich conditions. S1(hole)‐VS_2_‐VS had the lowest defect formation energy (1.0 to 1.7 eV), indicating superior stability. The structural stability of VS_2_‐VS and S1(hole)‐VS_2_‐VS at 300K was assessed using ab initio molecular dynamics (AIMD). Both structures maintained their superlattice integrity after 10 000 fs at 300K (Figure 3f,g), with energy fluctuations within −714 to −712 eV and −702 to −700 eV, respectively, showing no significant oscillations. As the temperature increases, the amplitude of energy fluctuations also increases, but both materials still maintain good thermal stability at 500K (Figure S21, Supporting Information). Finally, a comparison of V─S bond lengths (Figure S22 and Table S3, Supporting Information) revealed that the formation of a vacancy at the S1 position slightly altered the bond lengths, especially in the VS_2_ layer near the defect, but had minimal impact on the VS chain and heterogeneous interface. Overall, the lower concentration of S‐vacancy does not significantly affect the structural stability of the defect model.

By controlling the growth conditions, we successfully synthesized both the pure VS_2_‐VS superlattices and V_S_‐VS_2_‐VS defective materials with sulfur atomic vacancies. Herein, all superlattice materials were synthesized using a salt‐assisted chemical vapor deposition (CVD) method,^[^ ^48^, ^67^, ^68^ ^]^ with detailed experimental procedures provided in the synthetic methods section (supporting information). The corresponding defective structure was achieved by adjusting the precursor ratio between S and V.

**Figure 4:**
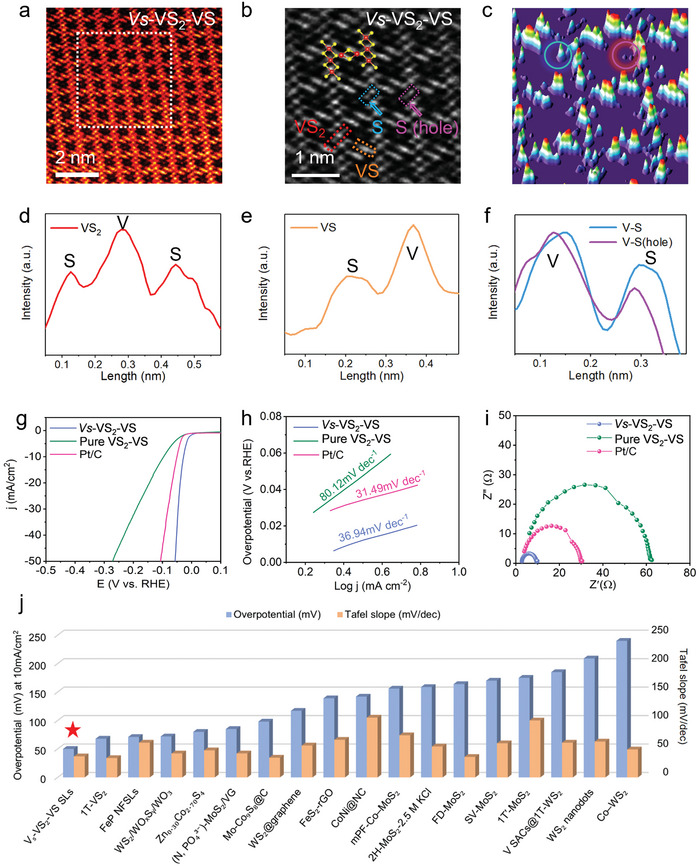
Structural characterization and HER performance of *V*_*S*_‐VS_2_‐VS. a) Cross‐section HAADF image of the *V*_*S*_‐VS_2_‐VS superlattice containing S point‐defects, with the overlaid atomic model structure. b) The enlarged atomic image from the white rectangle area of (a), the atomic model presents VS_2_ and VS atom structure, the red, yellow, blue, and pink dashed area presents VS_2_, VS, V─S bond, and V‐S (hole) bond respectively. c) The 3D atomic structure image from (b). d–f) The line profiles of VS_2_, VS, and V‐S with V‐S (hole) bond respectively. g,h). Polarization curves (g) and Tafel plots (h) of the *V*_*S*_‐VS_2_‐VS, VS_2_‐VS, and 20% Pt/C in 0.5 M H_2_SO_4_. i) Nyquist plots of *V*_*S*_‐VS_2_‐VS, VS_2_‐VS, and Pt/C. j) Overpotentials and Tafel slope at a current density of 10 mA·cm^−2^ of *V*_*S*_‐VS_2_‐VS in comparison to values reported previously for HER catalysts in acidic electrolyte.

To demonstrate the successful synthesis of sulfur‐vacancy defective structure, a comprehensive characterization of the corresponding materials' atomic structures was conducted. **Figure** **4**a shows a high‐angle annular dark field (HAADF) image of the *V*_*S*_‐VS_2_‐VS superlattice cross‐section, captured using a 300 kV aberration‐corrected scanning transmission electron microscope (STEM). The observed stacking sequence aligned well with the atomic model illustrated in Figure 1a. Some weak atomic contrast appeared in the spacing of the VS chains, which is due to the migrating VS chains within the material. Additionally, partial distortion of the crystal structure around these vacancies was observed, likely due to V─S bond breakage in the VS_2_ layer. To vividly present the *V*_*S*_ ‐VS_2_‐VS atomic structure, we enlarged the white rectangle area from Figure 4a and exhibited its atomic structure in Figure 4b. The atomic model shows the regular VS_2_ and VS_2_ atom structure the red and yellow dots presenting V and S atoms. We directly observed the S1(hole) in a pink dashed area with a lower contrast. In detail, we show the S1(hole) by 3D contrast image in Figure 4c. A lower contrast of the atom column signed by a pink circle confirmed the existence of the sulfur vacancy. There was a significant difference in atomic structure in the *V*_*S*_‐VS_2_‐VS superlattice. For comparison, Figure S23 (Supporting Information) shows a high‐magnification HAADF image of the pure VS_2_‐VS superlattice from the side view, where a highly ordered crystal structure is present. Precisely, in Figure 4d,e, the line profiles of the VS_2_ and VS, from the red and yellow dashed area in Figure 4b, were illustrated. Due to the atom number difference between S and V, the higher and lower peaks presented the V and S atom columns, respectively. The as‐obtained *V*_*S*_‐VS_2_‐VS possessed high crystal quality (Figure 4d,f) In addition, we compared the S atom intensity from the regular V─S bond and V─S (hole) bond in the blue and pink dashed area from Figure 4b, respectively. The intensity of S1(hole) was apparently smaller than the regular S intensities, also supporting the true existence of S1(hole).

The electrocatalytic HER performance for the *V*_*S*_‐VS_2_‐VS, VS_2_‐VS, and 20 wt% Pt/C were investigated using a three‐electrode system in acid media. Cyclic voltammetry (CV) scans of the *V*_*S*_‐VS_2_‐VS system were conducted between −0.05 and 0 V versus RHE (Figure S24, Supporting Information). The CVs show almost no hysteresis loops within the non‐Faradaic region, indicating that the double‐layer contribution is minimized for the configured samples. This implies that capacitive effects are negligible and do not significantly affect the HER performance in this case. This clarification ensures that the capacitive behavior has been carefully considered and excluded from influencing the HER performance evaluation. The electrochemical measurements were conducted with 90% iR correction and the LSV curves of *V*_*S*_‐VS_2_‐VS both before and after iR correction are shown in Figure S25 (Supporting Information). As depicted in Figure 4g, the *V*_*S*_‐VS_2_‐VS required an overpotential of 46 mV to achieve a current density of 10 mA·cm^−2^, demonstrating superior HER activity compared to Pt/C. In contrast, the pure VS_2_‐VS superlattice exhibited lower HER activity (96 mV). The Tafel slope of the *V*_*S*_‐VS_2_‐VS superlattice (36.94 mV·dec^−1^) was half of the value of is VS_2_‐VS superlattice (80.12 mV·dec^−1^), indicating the improved HER reaction kinetic induced by Vs engineering (Figure 4g). Accordingly, the pure VS_2_‐VS superlattice followed a typical Volmer‐Heyrovsky mechanism, with the Heyrovsky step, where an adsorbed hydrogen atom combines with a proton and electron to form H_2_, serving as the rate‐determining step. For the Volmer‐Tafel mechanism, two adsorbed hydrogen atoms recombined on the surface to form and release H_2_, generally exhibiting a Tafel slope ≈30–40 mV·dec^−1^. Thus, the Tafel slope value of *V*_*S*_‐VS_2_‐VS suggested a Volmer‐Tafel mechanism, with the recombination of active hydrogen as the rate‐determining step. This was also completely consistent with the conclusion obtained from our previous theoretical analysis of the HER mechanism of defective structures. With the sulfur vacancy, the electrochemical active surface area of *V*_*S*_‐VS_2_‐VS increased compared to that of VS_2_‐VS, indicating the sulfur vacancy favoring the increased number of active sites (Figure S26, Supporting Information).

Electrochemical impedance spectroscopy (EIS) was applied to investigate the electrode kinetics under HER operating conditions. As shown in Figure 4i, the small charge transfer resistance (Rct) value indicated that the *V*_*S*_‐VS_2_‐VS superlattice (10.5 Ω) have more favorable HER kinetics compared to 20% Pt/C (31.3 Ω) and VS_2_‐VS (62.8 Ω), which further suggests that defect engineering not only provides high active‐site but also facilitates the charge transfer efficiency. The EIS spectra were fitted according to the equivalent circuit model (Figure S27, Supporting Information), showing a significant decrease in Rct upon the introduction of sulfur vacancies. This trend is consistent with the changes in the Tafel slopes, further confirming that the introduction of sulfur vacancies improves electrode kinetics, thereby facilitating the charge transfer process between the electrolyte and the electrode surface. Specifically, the sulfur vacancies provide additional active sites on the electrode surface, increasing the availability of electronic states and thereby accelerating the charge transfer rate. The explanation of this mechanism offers deeper insight into the role of sulfur vacancies in enhancing catalytic performance.

The stability of the *V*_*S*_‐VS_2_‐VS heterodimensional superlattice was confirmed through long‐term testing after 15 000 cycles and constant overpotential testing over 10 h (Figures S28 and S29, Supporting Information). After 15 000 cycles, the η_10_ loss was less than 10 mV, and the initial current density was maintained without an obvious decrease after 10 h at a constant overpotential of 46 mV. These results support the excellent durability of the as‐prepared *V*_*S*_‐VS_2_‐VS superlattice. Moreover, the performance of *V*_*S*_‐VS_2_‐VS was superior to the most ever‐reported HER electrocatalysts in acid media (Figure 4j; Table S4, Supporting Information).

## 3 Conclusion

In summary, we designed a non‐noble metal sulfide electrocatalyst based on a VS_2_‐VS (2D‐1D) heterodimensional superlattice with sulfur vacancies, achieving a highly efficient hydrogen evolution reaction (HER). The outstanding catalytic performance of the VS_2_‐VS superlattice is primarily driven by the engineered heterojunction, where the work function difference between the VS_2_ layer and VS chain creates a charge separation field that effectively segregates electrons and holes, thereby enhancing charge separation. The introduction of sulfur vacancies further amplifies this effect by inducing quantum localization of the separated electrons, predominantly from V‐3*d* orbitals. This quantum localization improves hydrogen adsorption at the vacancy sites, optimizing the electronic environment and significantly enhancing HER activity. Theoretical calculations and experimental results confirmed that the heterodimensional superlattice achieves an overpotential of only 46 mV at 10 mA·cm^−2^ in acidic media, outperforming the HER activity of the 20% Pt/C catalyst while maintaining excellent electrochemical stability over 15 000 cycles. These results establish the VS_2_‐VS heterodimensional superlattice as a highly promising electrocatalyst for HER and offer important insights for the development of next‐generation catalysts aimed at sustainable energy applications.

## Conflict of Interest

The authors declare no conflict of interest.

## Author Contributions

E.S. and J.L. conceived the research. J.L. and W.D. contributed equally to this work. J.L carried out the theoretical calculations. Y.Y. and J.Z. conceived the synthetic scheme and carried out the materials preparation. W.D. and Y.Z. carried out the structural and electrochemical characterizations. J.L., E.S., Y.Z., and J.L. wrote the manuscript. All authors discussed the results and commented on the manuscript.

## Supporting information



Supporting Information

## Data Availability

The data that support the findings of this study are available from the corresponding author upon reasonable request.
