# Supplementary material for: Optimizing Interfacial Charge Dynamics and Quantum Effects in Heterodimensional Superlattices for Efficient Hydrogen Production
Source: Adv Sci (Weinh). 2024 Dec 16;12(6):2412805. doi: 10.1002/advs.202412805 (PMC11809332; doi:10.1002/advs.202412805)
Supplement: Supplementary file 1 — Supporting Information [file ADVS-12-2412805-s001.docx]

**Supporting Information**

**Optimizing Interfacial Charge Dynamics and Quantum Effects in Heterodimensional Superlattices for Efficient Hydrogen Production**

Jinpeng Li^#^, Weikang Dong^#^, Zibo Zhu, Yang Yang, Jiadong Zhou, Sufan Wang*, Yao Zhou*, Erhong Song*, Jianjun Liu*

J. Li, Prof. E. Song, Prof. J. Liu,

State Key Lab of High Performance Ceramics and Superfine microstructure, Shanghai Institute of Ceramics, Chinese Academy of Sciences, Shanghai 200050, China.

E-mail: ehsong@mail.sic.ac.cn; jliu@mail.sic.ac.cn

W. Dong, Y. Yang, Prof. J. Zhou, School of Physics, Beijing Institute of Technology, Beijing 100081, China.

Z. Zhu, School of Materials and Chemistry, University of Shanghai for Science and Technology, Shanghai 200093, P. R. China

Prof. S. Wang, College of Chemistry and Materials Science, Anhui Normal University, Wuhu 241000, P. R. China.

E-mail: sfwang@ ahnu.edu.cn

Prof. Y. Zhou, Advanced Research Institute of Multidisciplinary Science, Beijing Institute of Technology, Beijing 100081, China.

E-mail: zhouyao@bit.edu.cn

**Calculation methods**

The Vienna Ab initio Simulation Package was used to perform spin-polarized density functional theory (DFT) calculations.^[^[^1^](#_ENREF_1)^]^ The Perdew-Burke-Ernzerhof (PBE) generalized gradient approximation was employed to describe electron exchange and correlation effects.^[^[^2-3^](#_ENREF_2)^]^ A plane-wave cutoff energy of 400 eV was applied, and the projector augmented wave (PAW) method was used to account for electron-ion interactions.^[^[^4^](#_ENREF_4)^]^ To incorporate long-range van der Waals interactions, the Grimme DFT-D2 dispersion correction was used.^[^[^5^](#_ENREF_5)^]^ He localized d orbitals of vanadium atoms were treated with Dudarev et al.’s approach using an effective U value of 3.0 eV.^[^[^6^](#_ENREF_6)^]^ For geometric optimization, a (2 × 12 × 2) k-point mesh was used, with convergence criteria set at 10^−5^ eV in energy and 0.01 eV/Å in force. A denser k-point mesh of (4 × 24 × 4) was adopted for electronic structure calculations to improve accuracy. A VS_2_-VS structure containing 52 atoms was modeled. The free energy of the adsorbed state was calculated as:

ΔG=ΔE_H*_ +ΔE_ZPE_－TΔS

where ΔE_H*_ is the hydrogen chemisorption energy, and ΔE_ZPE_ is the zero point energy difference between the adsorbed state and the gas phase. Given the minimal vibriation entropy of H* in the adsorbed state, the entropy of 1/2 H_2_ adsorption is approximated as ΔS_H_ ≈ −1/2 $S_{H^{2}}^{0}$ , where $S_{H^{2}}^{0}$ is the entropy of H_2_ gas understand standard conditions.

The acidic HER has two mechanisms, as below:

Volmer-Heyrovsky mechanism:

1. Volmer step: H^+^ + * + e^₋^ →H*
2. Heyrovsky step: H* + H^+^ + e^₋^ → H_2_

Volmer-Tafel mechanism:

1. Volmer step: H^+^ + * + e^₋^ →H*
2. Tafel step: H* + H*→ H_2_

where e^₋^ and * represent the electron and active site, respectively. H* is the adsorbing H atom on active site. Volmer step occurs in series with the Heyrovsky and Tafel steps, while the Heyrovsky and Tafel steps occur in parallel with each other.

**Synthesis of VS_2_-VS and *V_S_*-VS_2_-VS heterodimensional superlattice**

The synthetic processes for VS_2_-VS and *V_S_*-VS_2_-VS heterodimensional superlattices are largely consistent with previous research.^[^[^7-8^](#_ENREF_7)^]^ In a typical synthesis, a mixture of V_2_O_5_ and KI in a 10:1 ratio was used as the precursor, placed at the center of an alumina boat in a tube furnace. SiO_2_/Si substrates were positioned on the same boat. Sulfur (S) powder was placed upstream in the tube furnace at 200 °C. A hydrogen/argon gas mixture (10/80 standard cubic centimeters per minute) was used as the carrier gas. The growth temperature was set to 800 °C for 5 minutes with a ramping rate of 50 °C per minute. The ratio of sulfur to V_2_O_5_ was adjusted to obtain different products: a 5:1 ratio produced *V_S_*-VS_2_-VS, while an 8:1 ratio yielded VS_2_-VS.

**Structural and Electrochemical Characterizations**

**FIB Experiment and STEM Data Acquisition.**

The *V_S_*-VS_2_-VS sample was prepared using a ThermoFisher Helios G4 UC double-beam microscope. To prevent surface irradiation damage during milling, the sample was coated with 20 nm thick carbon layer followed by a 1 um thick platinum film. The final sample thickness was reduced to 80 nm. Structural imaging was carried out using a ThermoFisher Themis Z TEM equipped with double aberration correctors, operating at 300 kV for scanning transmission electron microscopy (STEM) in high-angle annular dark-field (HAADF) mode. The convergence angle was set to 25.2 mrad, and the screen current was 0.05 nA. Data was processed using ThermoFisher Velox software.

**Electrochemical Characterizations.**

Electrochemical tests were performed using heterodimensional superlattice catalysts deposited on the SiO_2_/Si substrates. Poly methyl methacrylate (PMMA) dissolved in methylbenzene was uniformly spun onto the substrate and baked at 80 °C for 5 minutes. The PMMA films covered substrates were then immersed in a 5 M KOH solution to etch away the SiO_2_/Si substrate. The detached heterodimensional superlattice catalysts/PMMA films were washed in deionized (DI) water and transferred onto a glassy carbon rotating disk electrode (RDE). The PMMA films were subsequently removed by immersing the samples in acetone, leaving the heterodimensional superlattice catalysts on the RDE. Electrochemical measurements were carried out in 0.5 M H_2_SO_4_ aqueous solution using a standard three-electrode setup, with the glassy carbon RDE as the working electrode, graphite carbon as the counter electrode, and a saturated calomel electrode (SCE) as the reference electrode. The potential versus the reversible hydrogen electrode (RHE) was calculated as:

*E*_RHE_ = *E*_SCE_ + *E*°_SCE_ (0.2412) + 0.059 × pH

Linear sweep voltammetry (LSV) measurements were conducted with a scan rate of 2 mV s^−1^ under 1500 rpm. Electrochemical impedance spectroscopy (EIS) was obtained in the frequency range of 100 KHz to 0.1 Hz at an applied current of 10 mA cm^−2^. In addition, long-term stability was evaluated by measuring the constant current 10 mA cm^−2^.


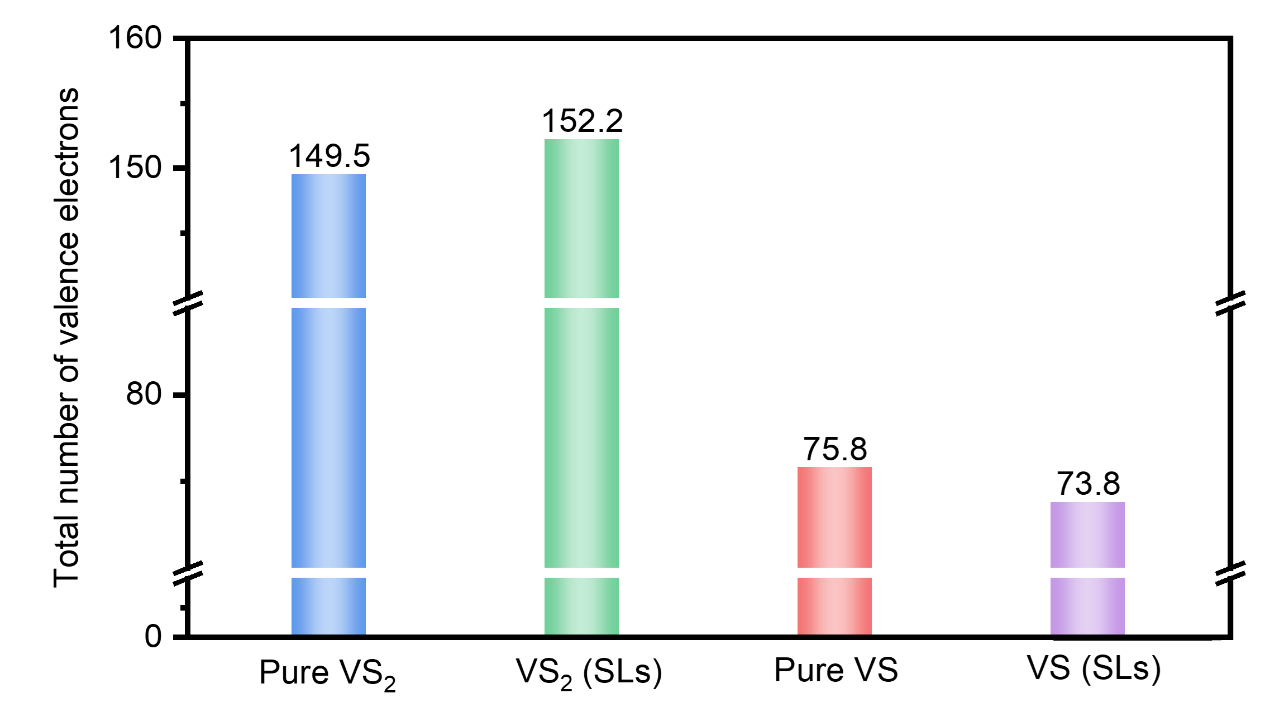


**Figure S1.** Total number of valance electrons (sum of valence electrons of V and S atoms) of pure VS_2_, pure VS and VS_2_-VS superlattices (SLs).


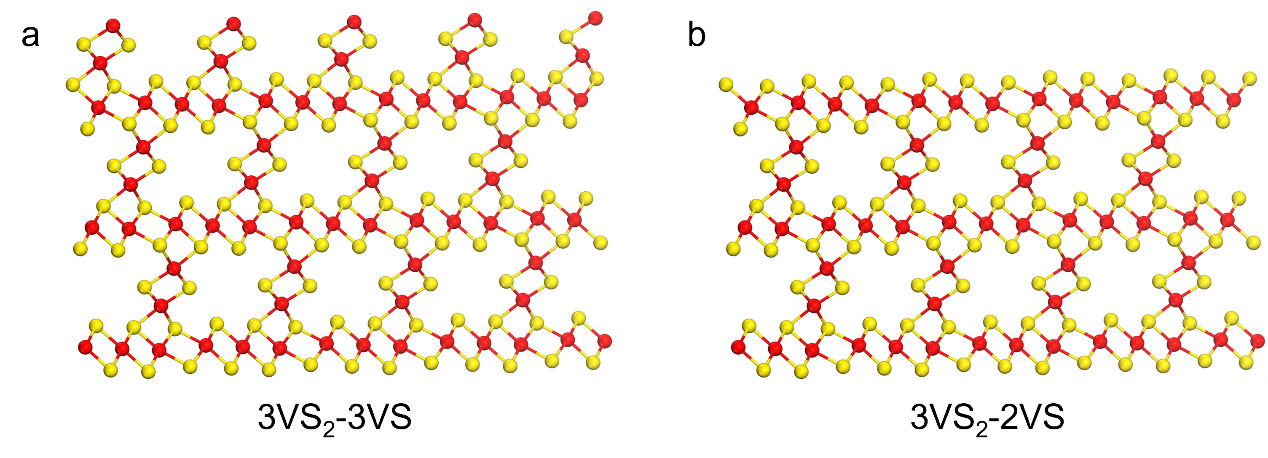


**Figure S2. a-b**, The two slab models of VS_2_-VS: 3VS_2_-3VS (a) and 3VS_2_-2VS (b).


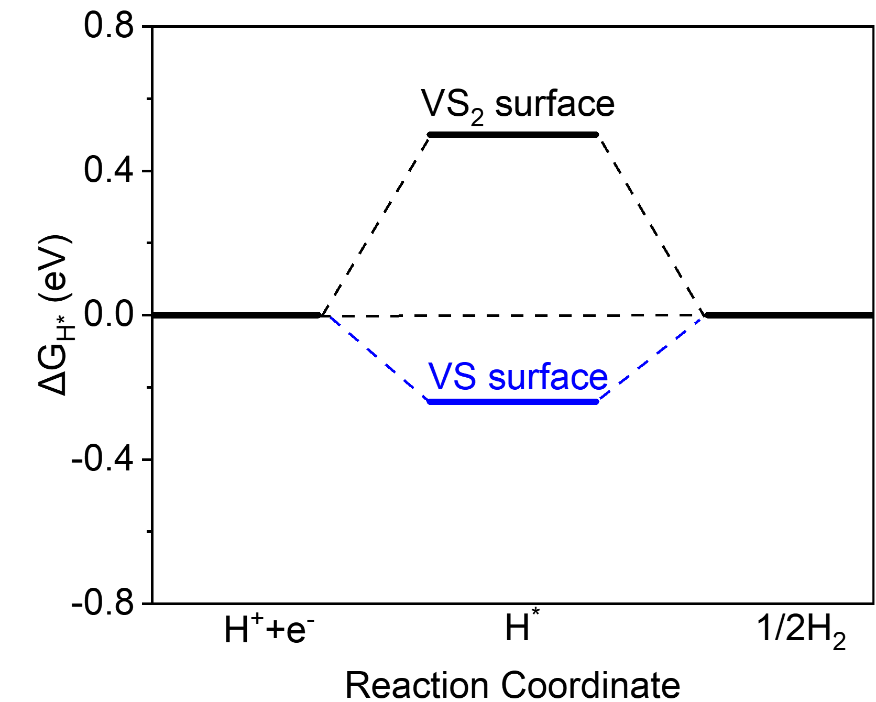


**Figure S3.** The calculated ΔG_H*_ of VS_2_ surface, VS surface and VS_2_-VS superlattice.


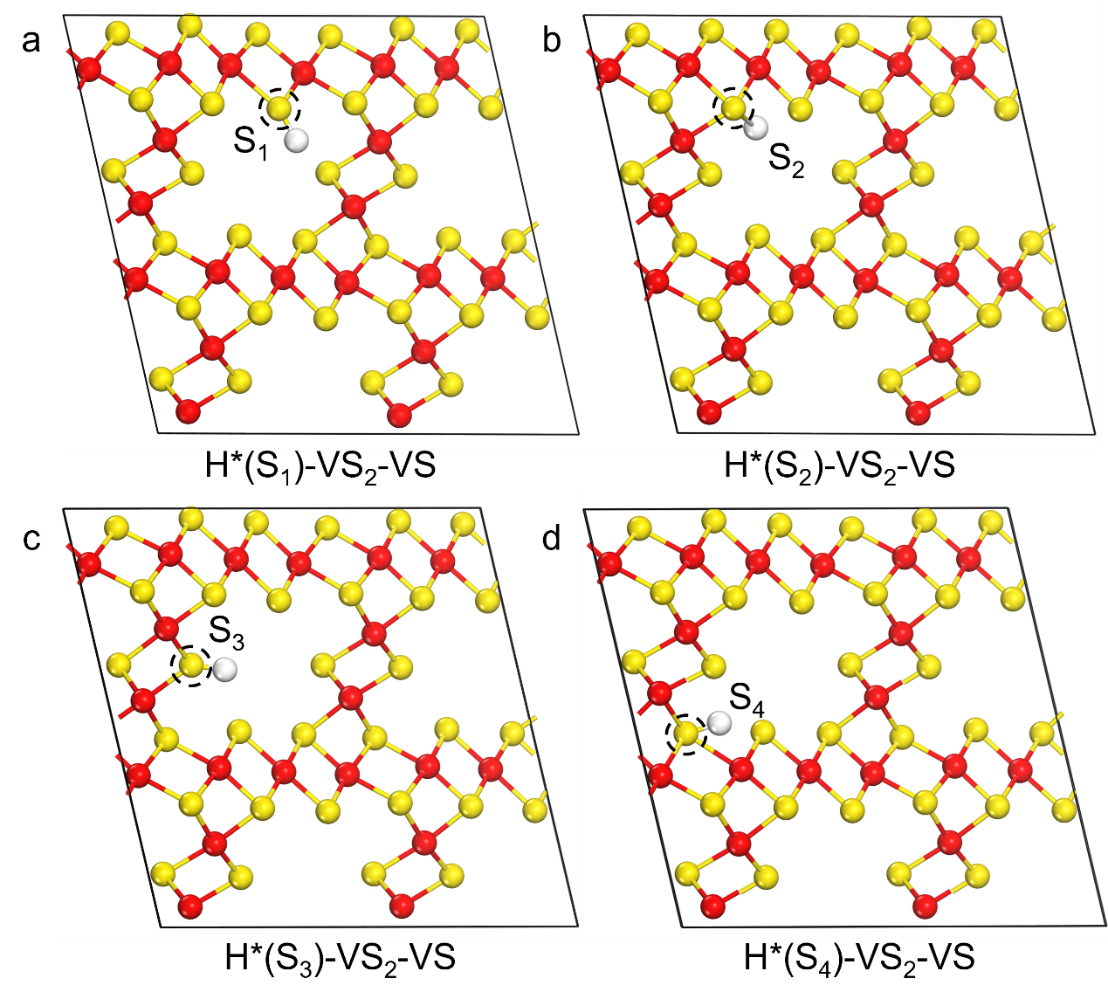


**Figure S4.** **a-d**, Side view of hydrogen adsorption configurations of VS_2_-VS superlattice.


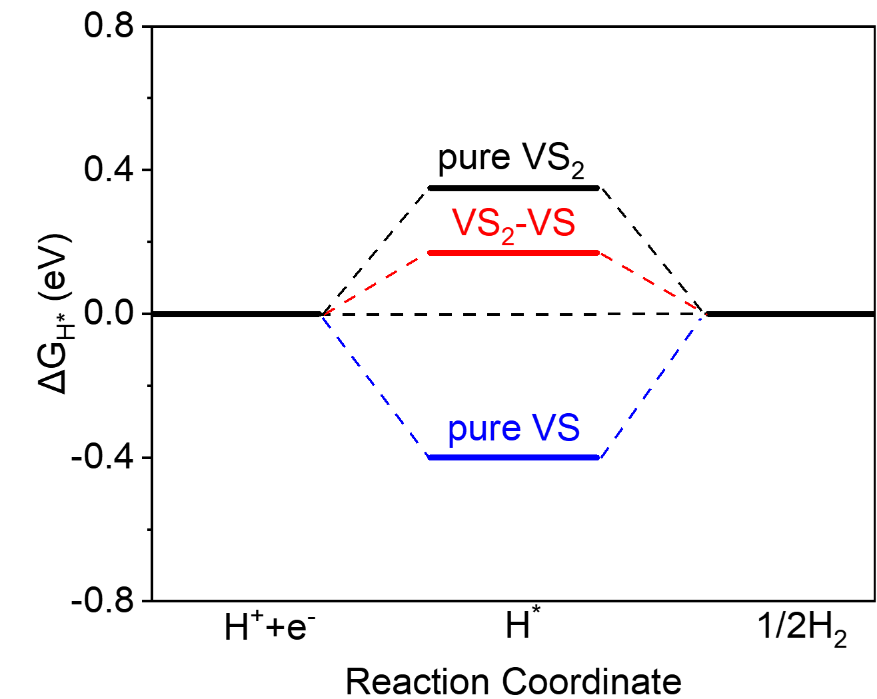


**Figure S5.** The calculated ΔG_H*_ of VS_2_ layer, VS chain and VS_2_-VS superlattice.


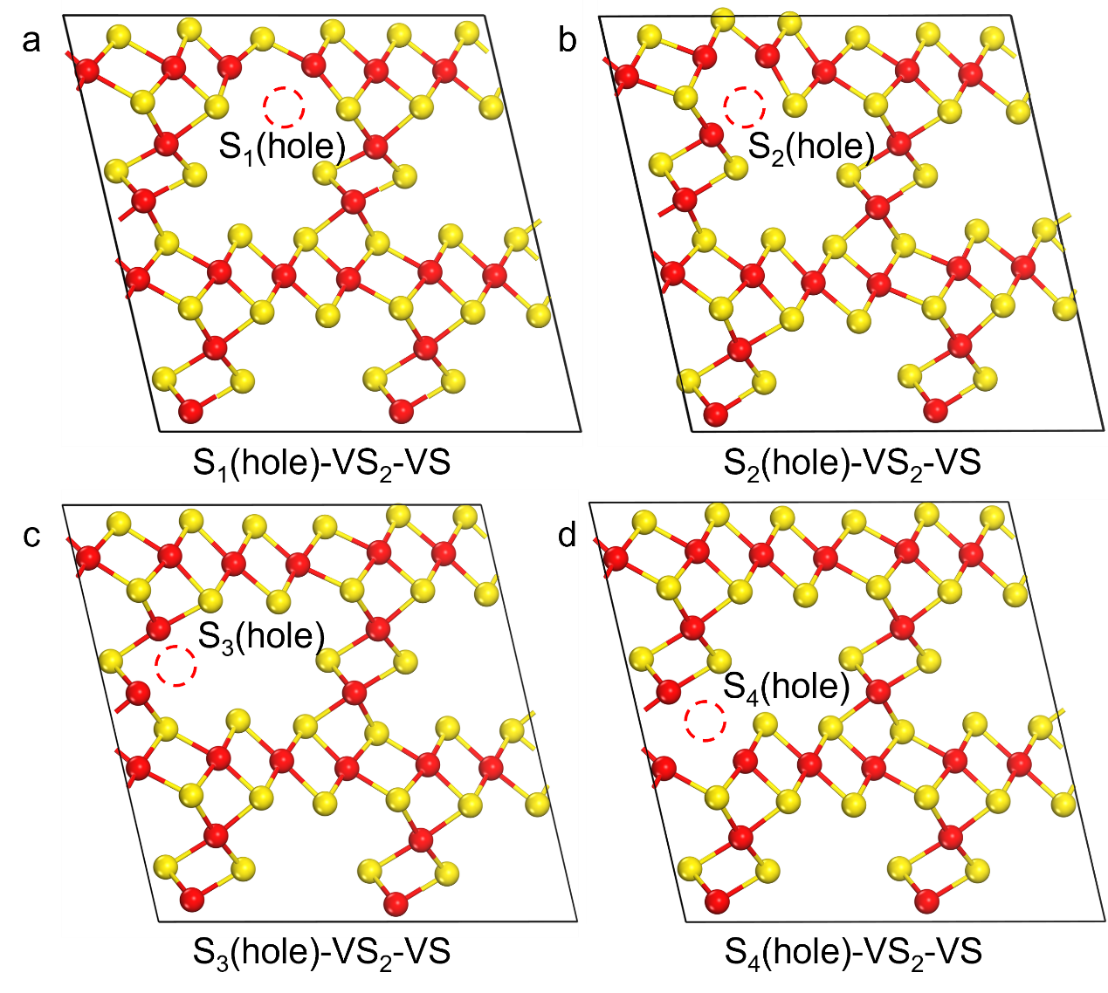


**Figure S6.** **a-d**, Side view of four kinds of S-defect models of VS_2_-VS superlattice.


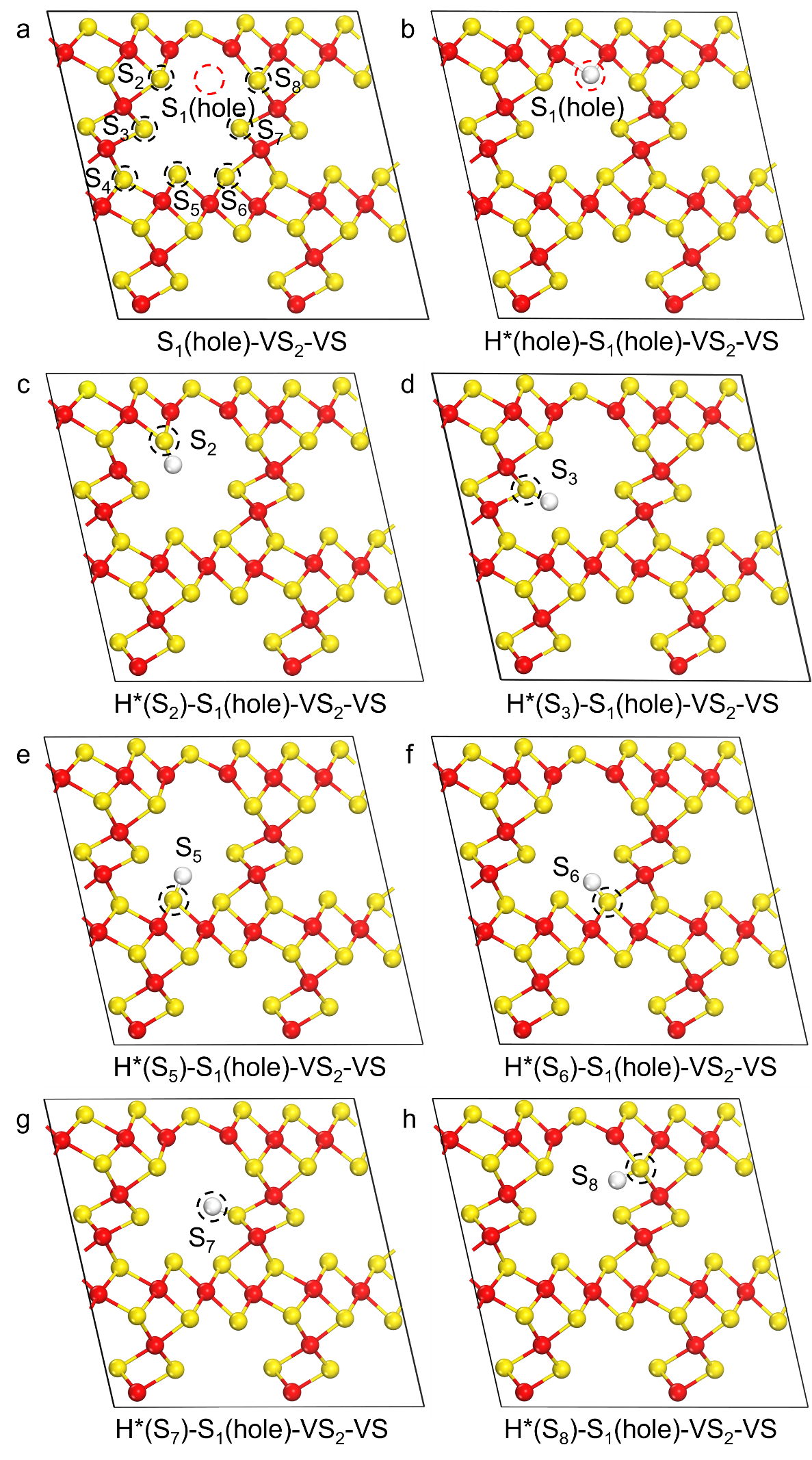


**Figure S7.** **a-h**, Side view of all possible adsorption sites (**a**) and hydrogen adsorption configurations (**b-h**) of S1(hole)-VS_2_-VS.


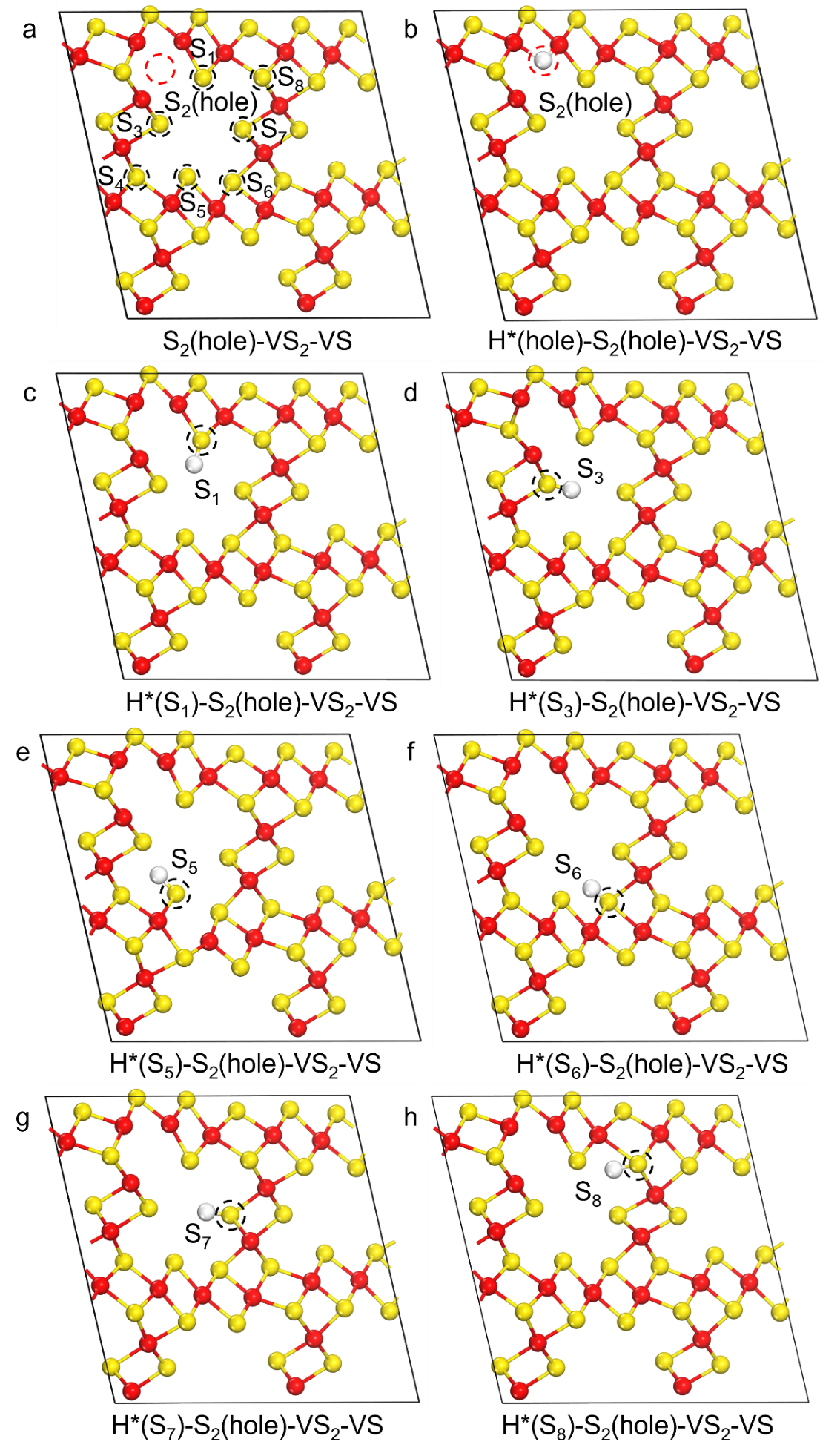


**Figure S8. a-h**, Side view of all possible adsorption sites (**a**) and hydrogen adsorption configurations (**b-h**) of S2(hole)-VS_2_-VS.


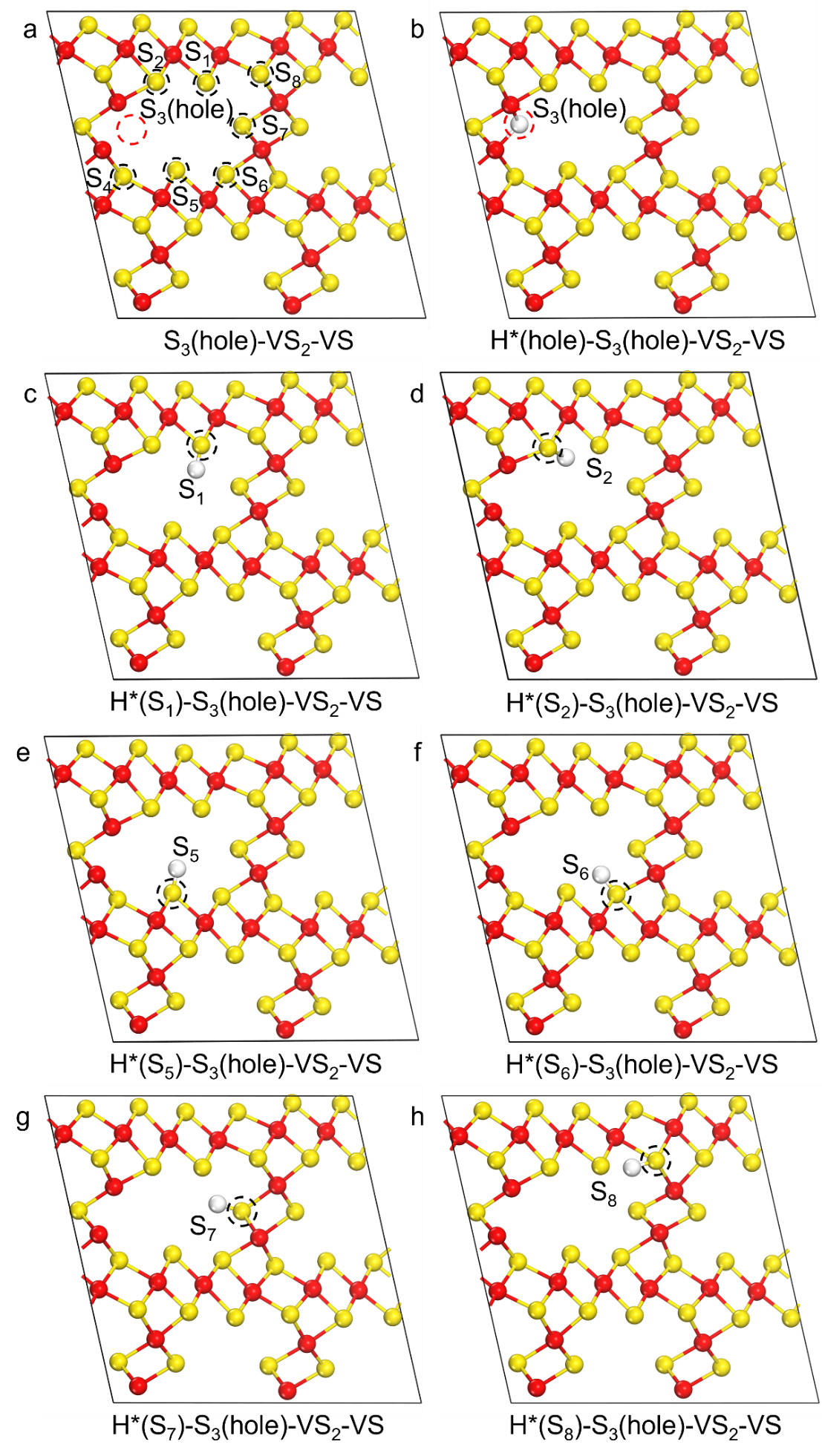


**Figure S9. a-h**, Side view of all possible adsorption sites (**a**) and hydrogen adsorption configurations (**b-h**) of S3(hole)-VS_2_-VS.


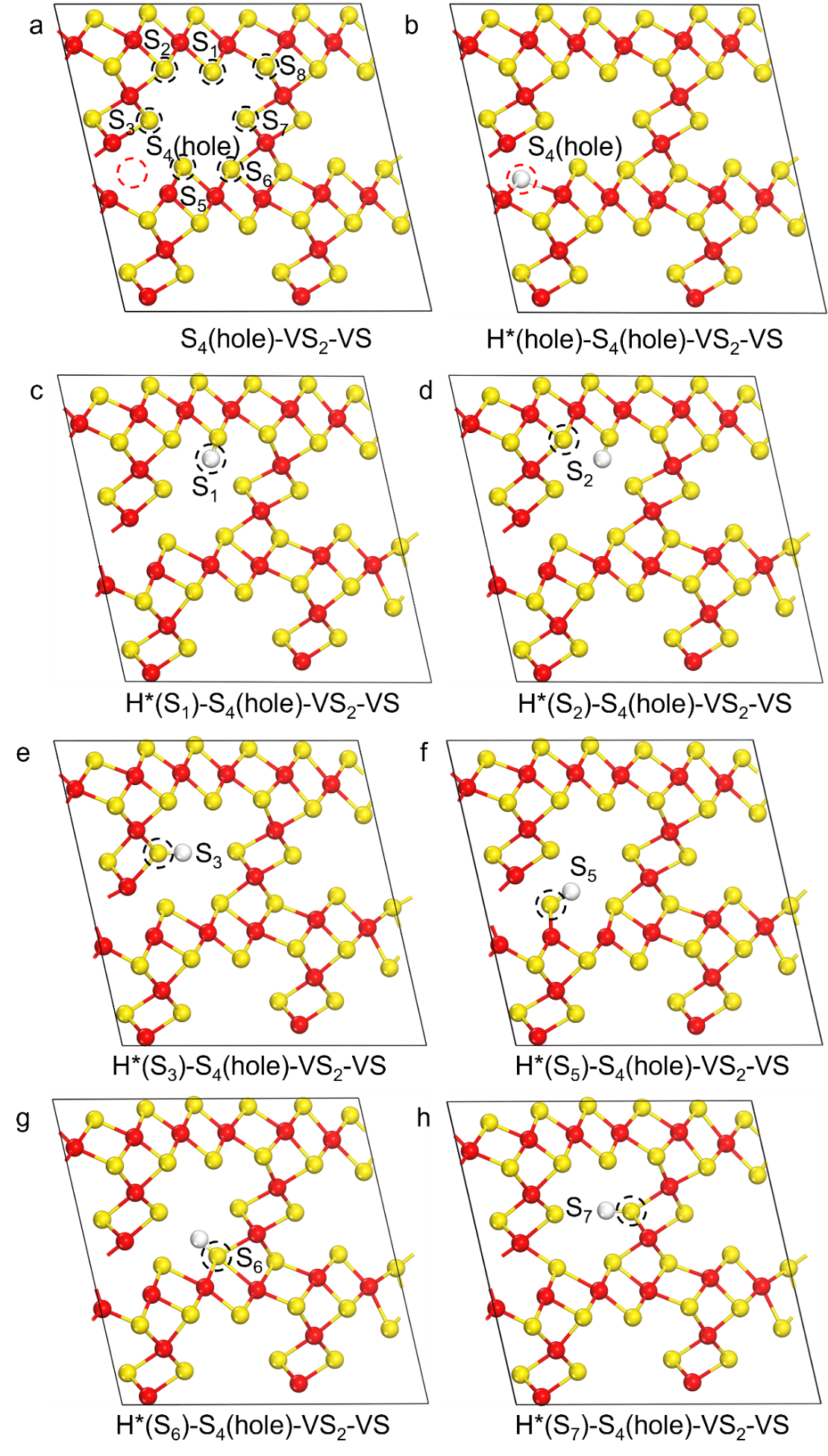


**Figure S10. a-h**, Side view of all possible adsorption sites (a) and hydrogen adsorption configurations (b-h) of S4(hole)-VS_2_-VS.


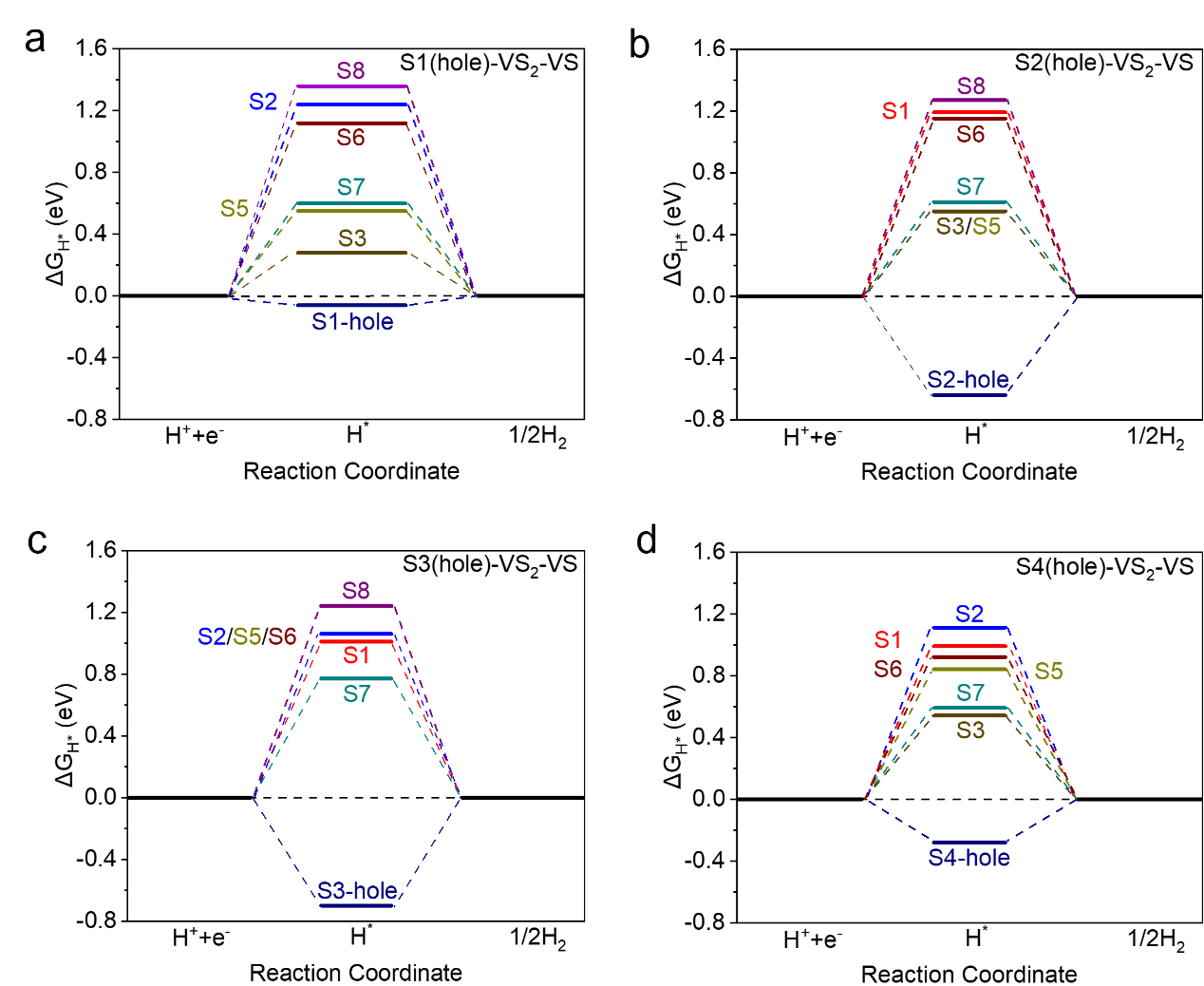


**Figure S11. a-d**, The calculated ΔG_H*_ of S-vacancy and all sulfur sites of four S-defect models in HER.


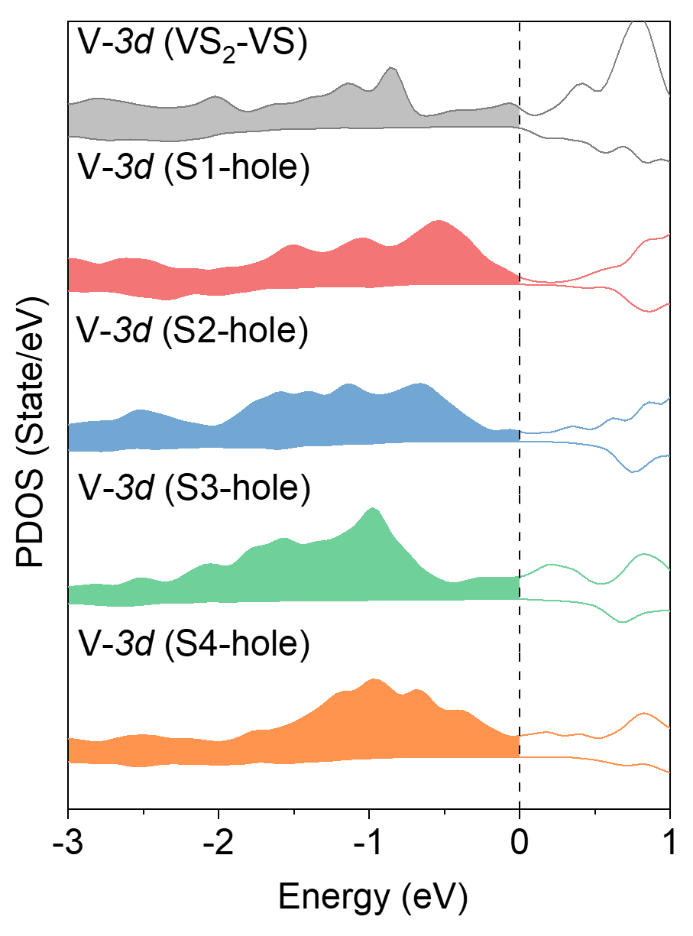


**Figure S12.** The projected density of states on V-*3d* orbitals for VS_2_-VS and four S-defect models.


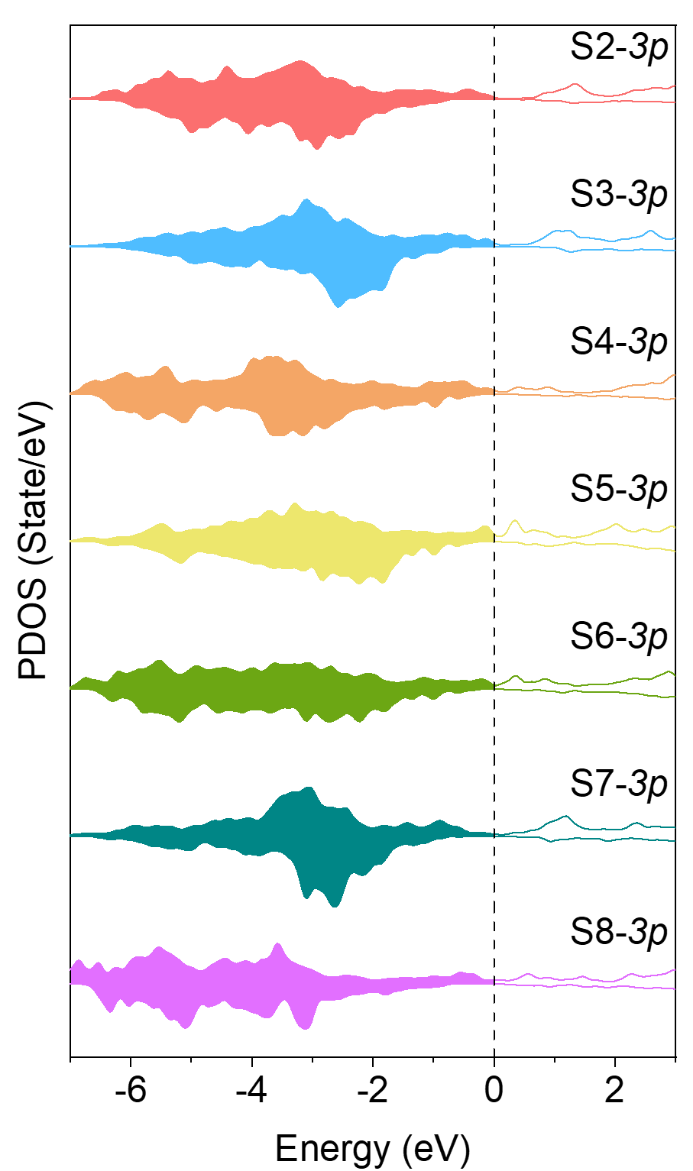


**Figure S13.** The projected density of states on S-*3p* orbitals for S1(hole)-VS_2_-VS.


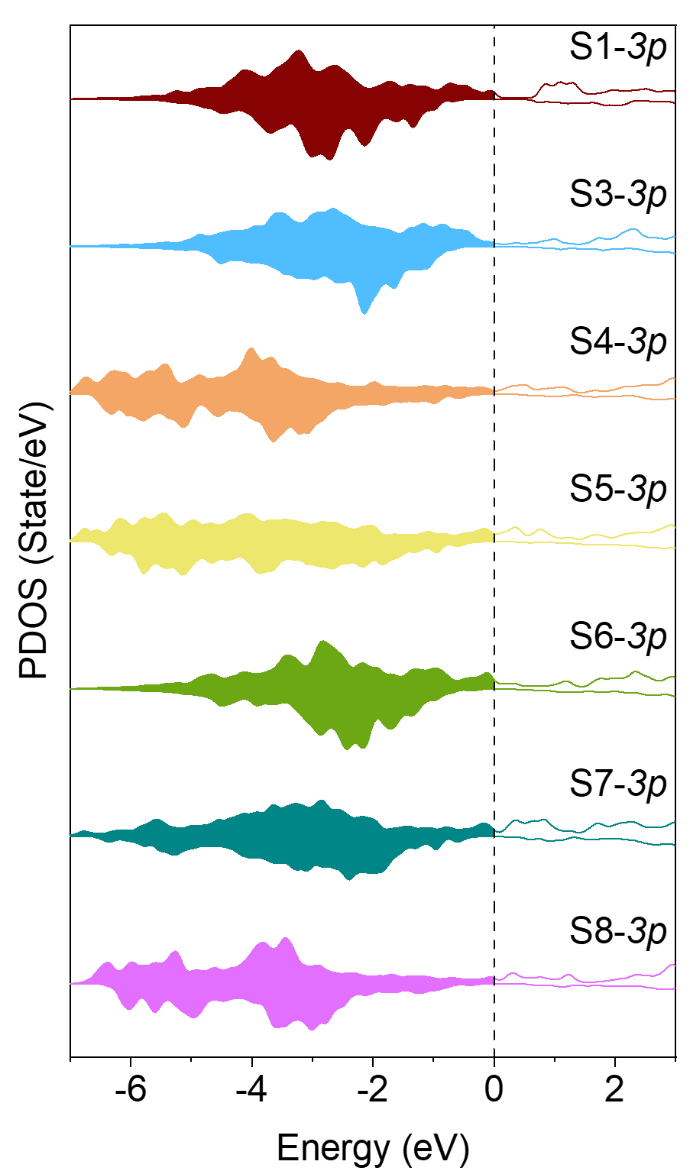


**Figure S14.** The projected density of states on S-*3p* orbitals for S2(hole)-VS_2_-VS.


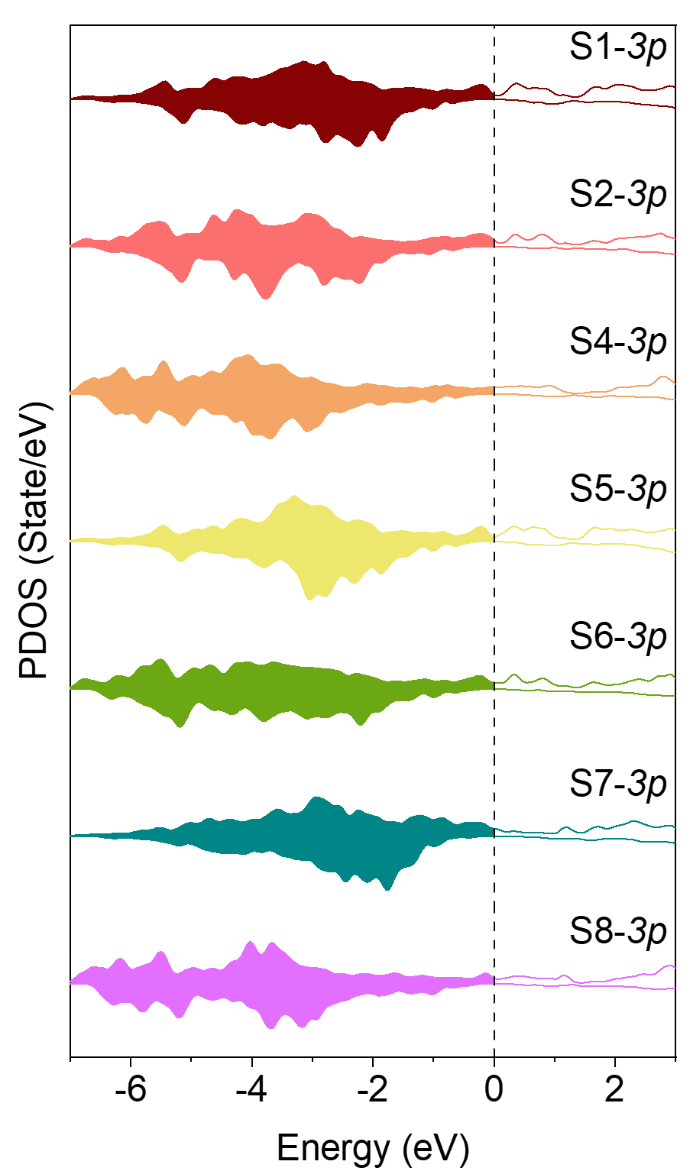


**Figure S15.** The projected density of states on S-*3p* orbitals for S3(hole)-VS_2_-VS.


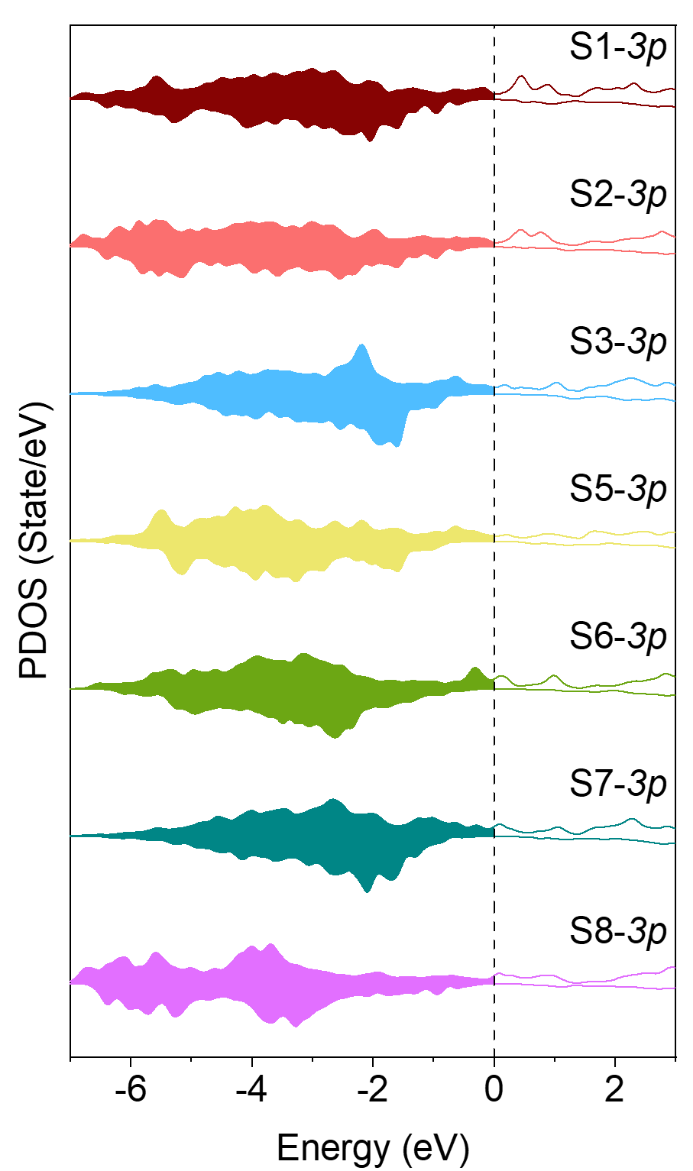


**Figure S16.** The projected density of states on S-*3p* orbitals for S4(hole)-VS_2_-VS.

**Figure S17.** Charge density difference plots of H*-S1(hole)-VS_2_-VS.


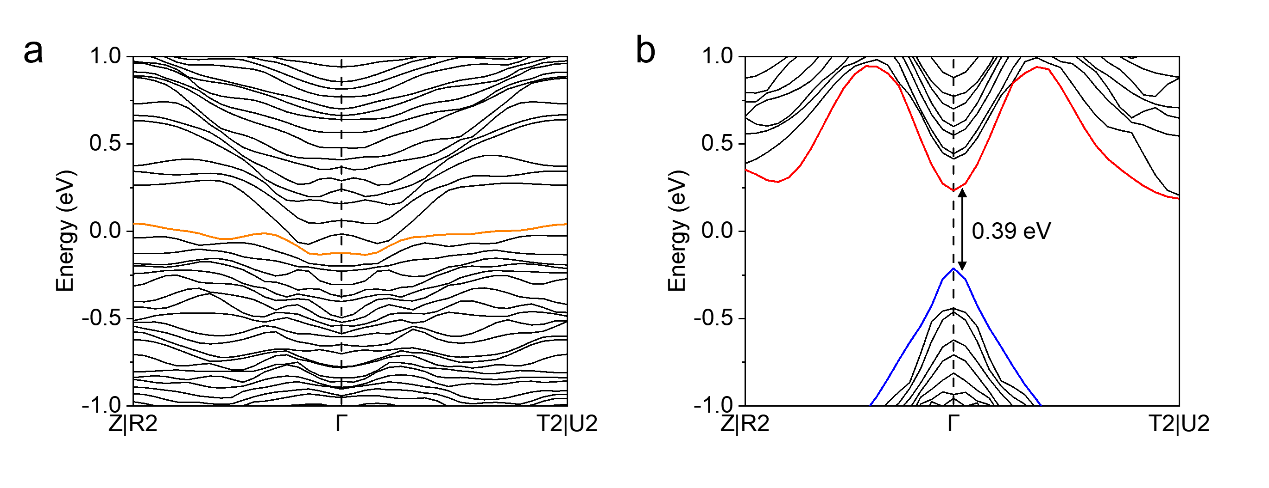


**Figure S18. a-b**, The spin-up (a) and spin-down (b) band structure for *V_S_*-VS_2_-VS as calculated with the HSE functional.


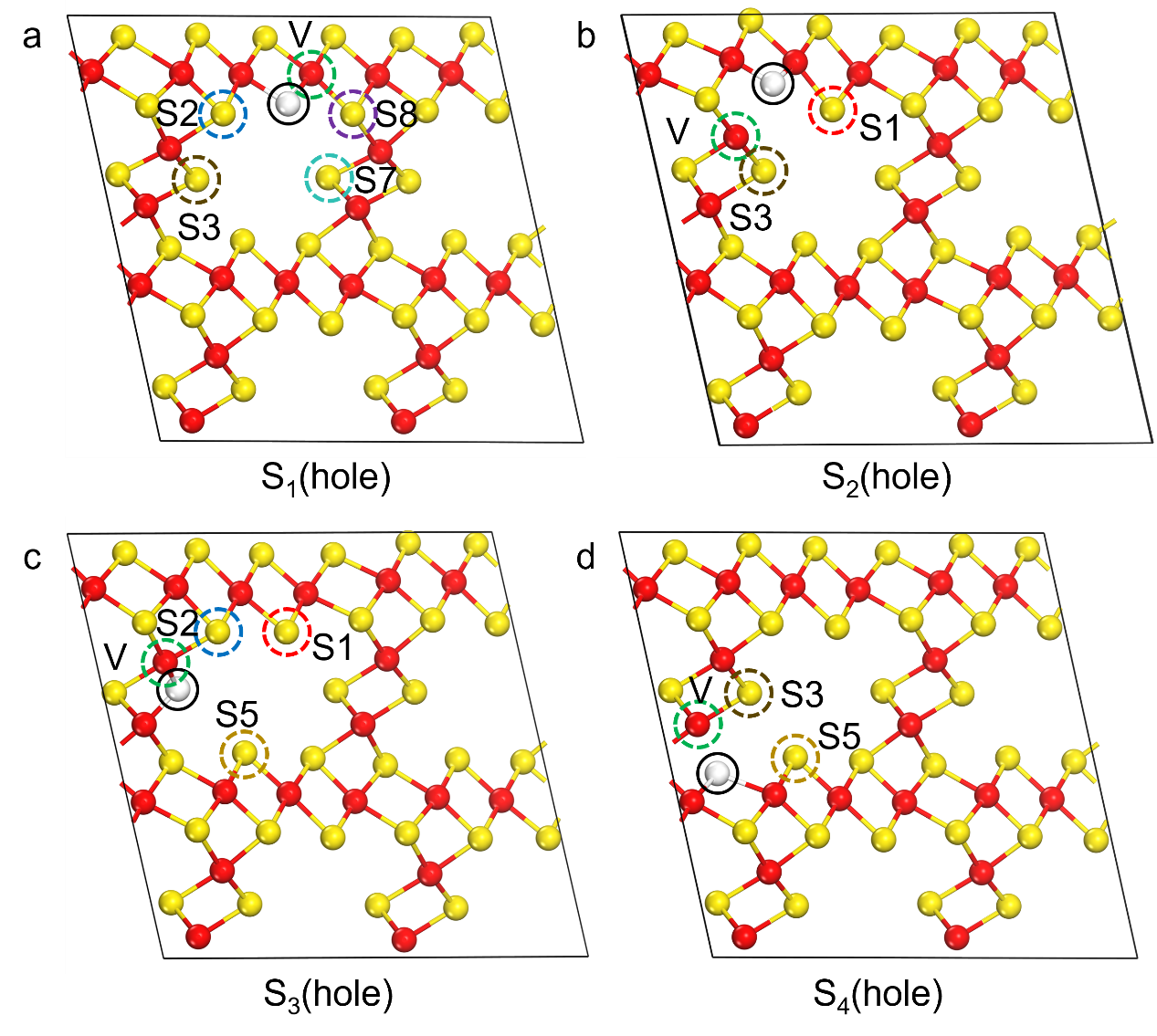


**Figure S19. a-d**, Black circles indicate the S-vacancies for hydrogen adsorption occurs during the Volmer step, while the remaining dashed circles indicate possible sites for hydrogen adsorption during the Tafel step.


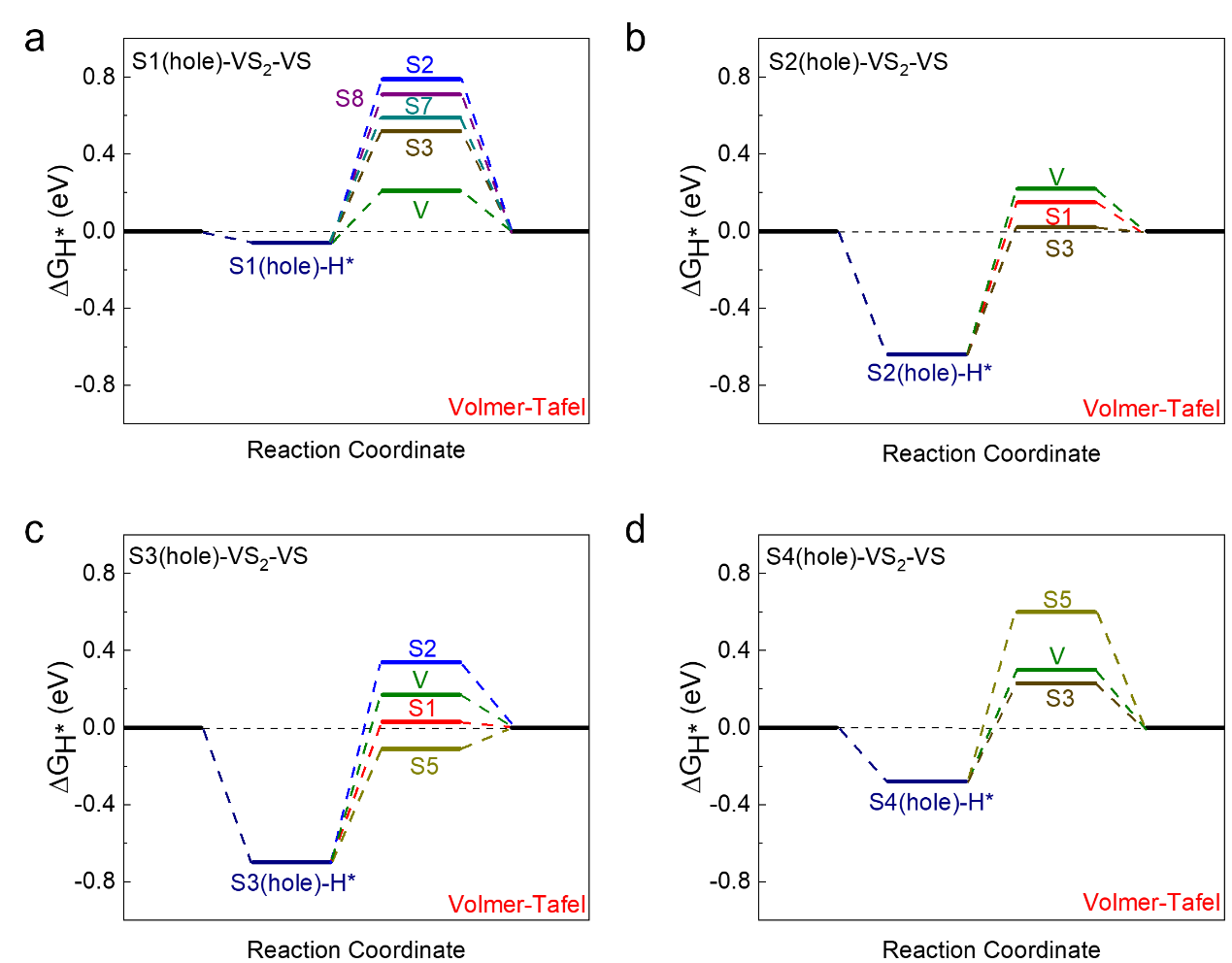


**Figure S20. a-d**, The calculated ΔG_H*_ of different sites for Tafel steps of S1(hole)-VS_2_-VS, S2(hole)-VS_2_-VS, S3(hole)-VS_2_-VS and S4(hole)-VS_2_-VS in HER.


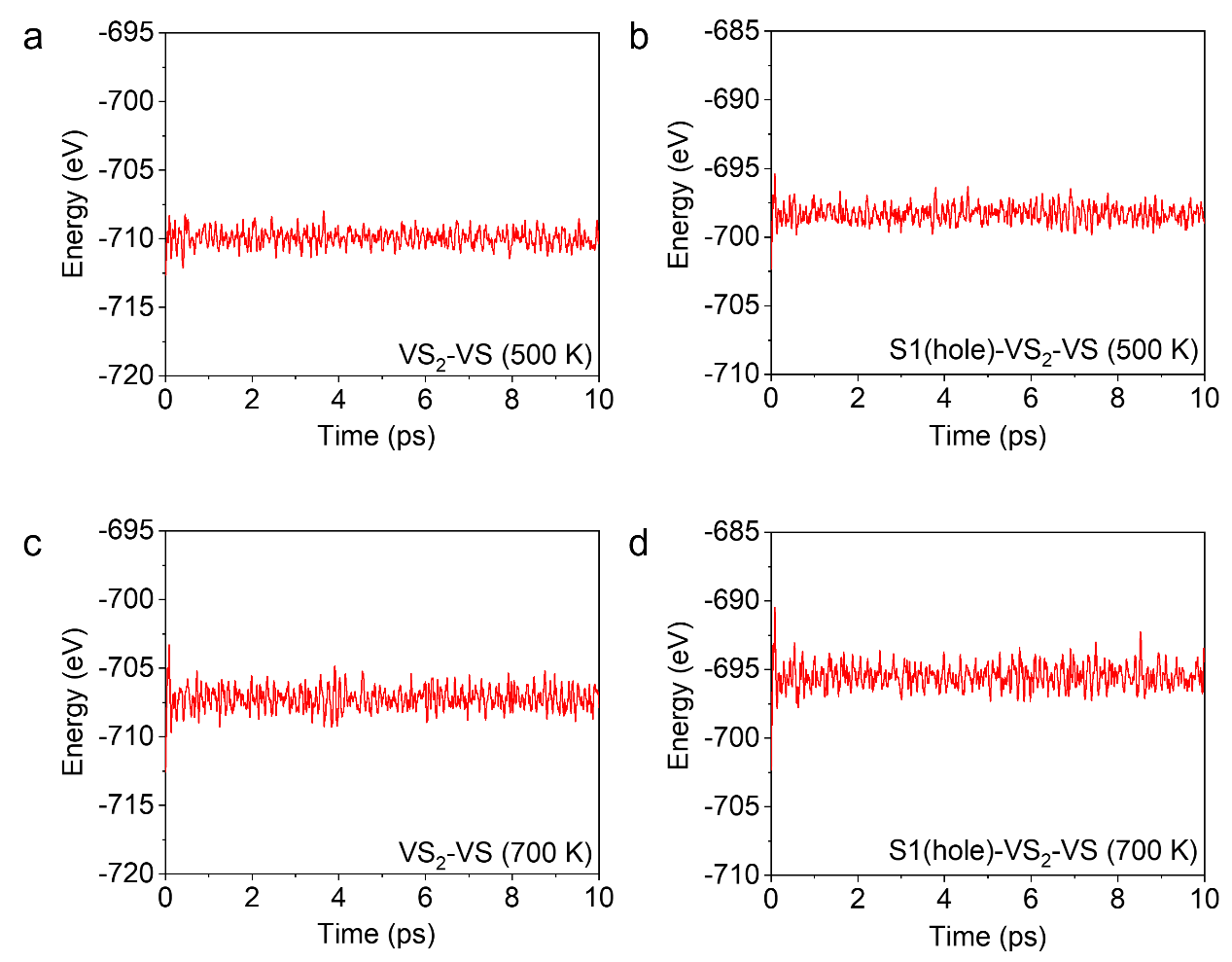


**Figure S21. a-d**, The energy fluctuation with time step at 500 K (a, b) and 700 K (c, d) of VS_2_-VS and S1(hole)-VS_2_-VS from AIMD calculation.


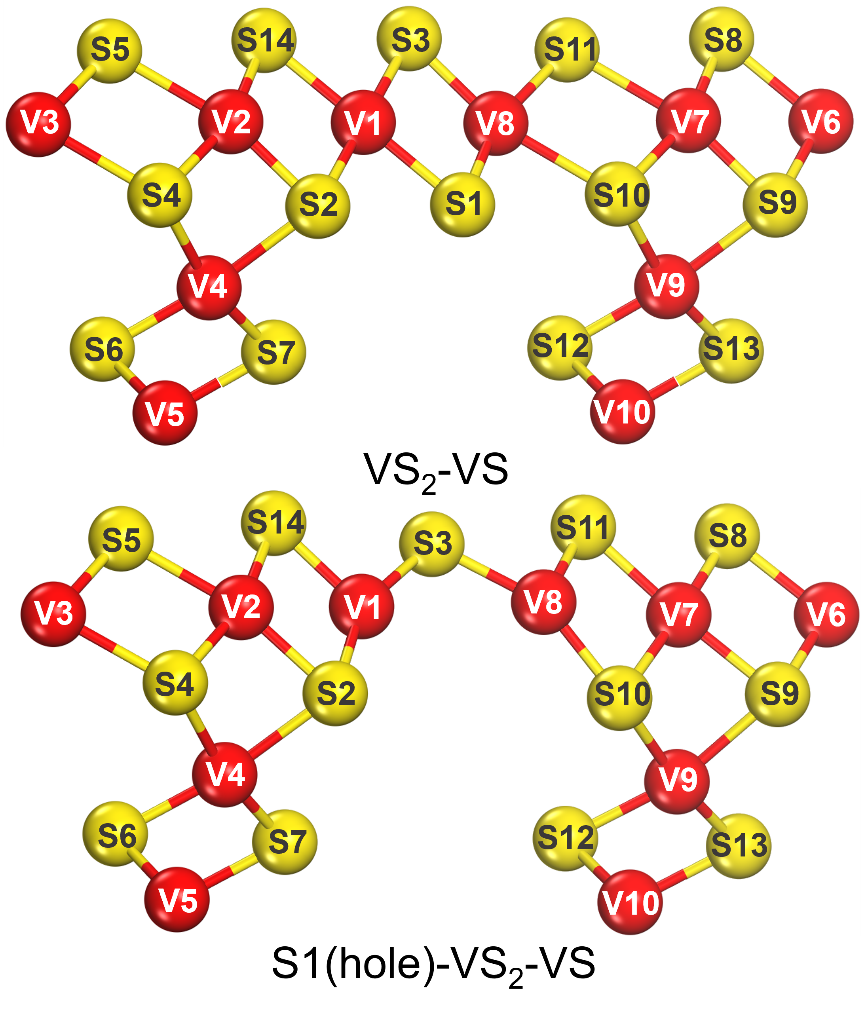


**Figure S22.** Partial structure diagram of VS_2_-VS and S1(hole)-VS_2_-VS with atomic numbers.


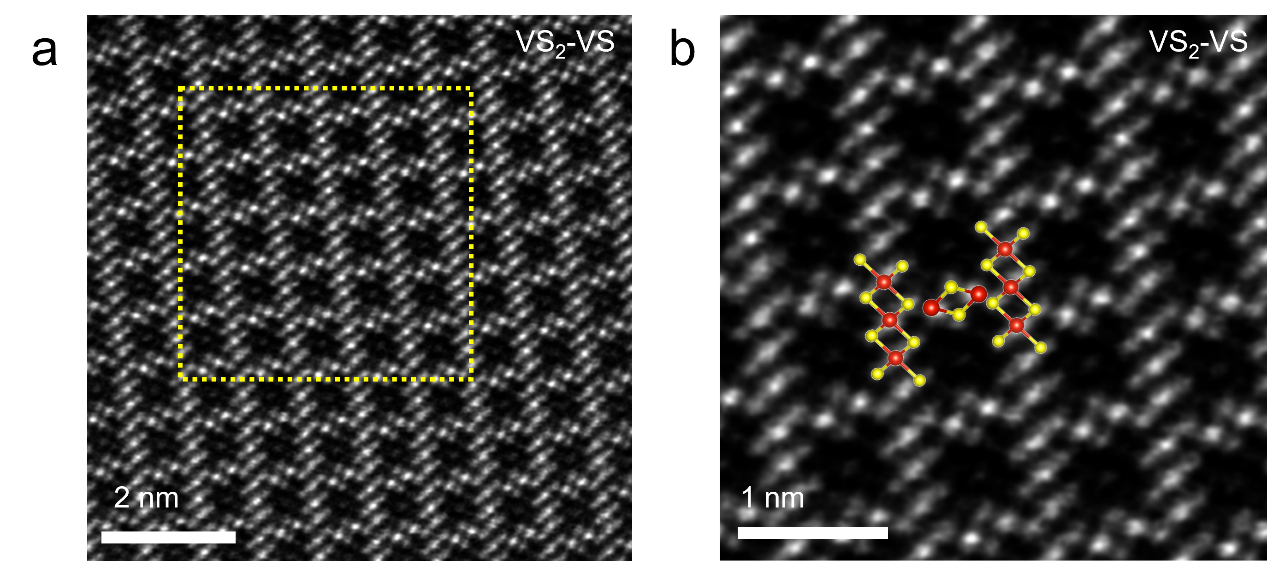


**Figure S23.** **a**, Cross-section HAADF image of the VS_2_-VS superlattice with the overlaid atomic model structure. **b**, The enlarged atomic image from the yellow rectangle area of (**a**).


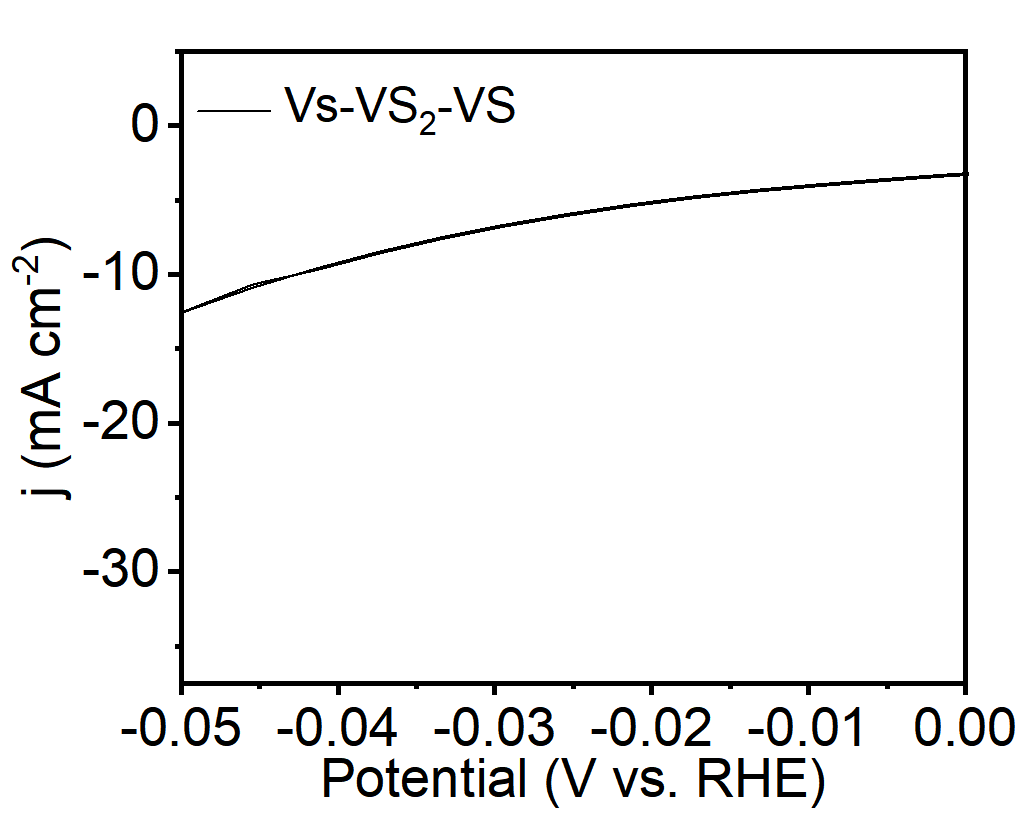


**Figure S24.** The CVs of the *V_S_*-VS_2_-VS sample for the HER between −0.05-0 V vs. RHE.


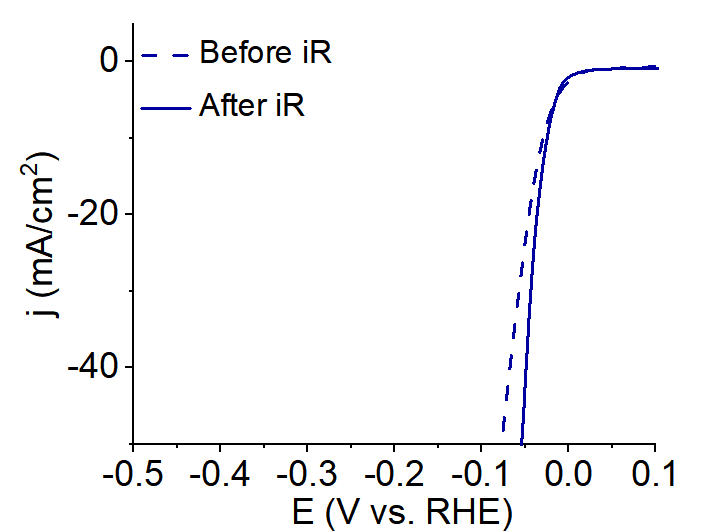


**Figure S25.** The LSV curves of *V_S_*-VS_2_-VS before and after iR corrections.

**Figure S26.** The measurements of the electrochemical active surface area of heterodimensional superlattice catalysts. The cyclic voltammetry curves with the scan rates from 40 to 160 mV s^−1^ for (**a**) Pt/C, (**b**) VS_2_-VS, and (**c**) *V_S_*-VS_2_-VS. (**d**) The double layer current density vs. scan rates plots of Pt/C, VS_2_-VS, and *V_S_*-VS_2_-VS.


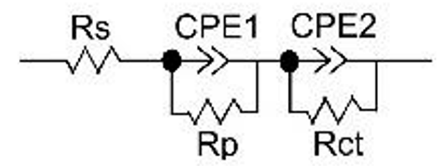


**Figure S27.** The circuit model for fitting electrochemical impedance spectroscopy.

**Figure S28.** The stability testing of *V_S_*-VS_2_-VS after 15000 cycles.

**Figure S29.** Long-test stability of the *V_S_*-VS_2_-VS electrode at the constant overpotential of 46 mV.

**Table S1.** The d-band center of VS_2_-VS, S1(hole)-VS_2_-VS and H*-S1(hole)-VS_2_-VS.

|  | V-*3d* /eV |
| --- | --- |
| VS_2_-VS | −2.13 |
| S1(hole)-VS_2_-VS | −1.76 |
| H*-S1(hole)-VS_2_-VS | −1.83 |

**Table S2.** Atomic charges based on Bader analysis of VS_2_-VS and S1(hole)-VS_2_-VS (the structure corresponds to Figure S22).

|  | Bader charge | loss/gain of e^−^ |
| --- | --- | --- |
| V1 (VS_2_-VS) | 11.57 | −1.43 |
| V8 (VS_2_-VS) | 11.59 | −1.41 |
| V1 (S1(hole)-VS_2_-VS) | 11.72 | −1.28 |
| V8 (S1(hole)-VS_2_-VS) | 11.82 | −1.18 |

**Table S3.** Comparison of bond lengths between VS_2_-VS and S1(hole)-VS_2_-VS (the structure corresponds to Figure S22).

|  |
| --- |

| **bond** | **VS_2_-VS(Å)** | **S1(hole)-VS_2_-VS(Å)** | **bond** | **VS_2_-VS(Å)** | **S1(hole)-VS_2_-VS(Å)** |
| --- | --- | --- | --- | --- | --- |
| V1-S1 | 2.378 | / | V6-S8 | 2.375 | 2.323 |
| V1-S2 | 2.385 | 2.330 | V6-S9 | 2.385 | 2.368 |
| V1-S3 | 2.387 | 2.374 | V7-S8 | 2.285 | 2.352 |
| V1-S14 | 2.374 | 2.220 | V7-S9 | 2.235 | 2.326 |
| V2-S2 | 2.236 | 2.337 | V7-S10 | 2.477 | 2.486 |
| V2-S4 | 2.477 | 2.456 | V7-S11 | 2.589 | 2.374 |
| V2-S5 | 2.588 | 2.536 | V8-S1 | 2.288 | / |
| V2-S14 | 2.284 | 2.324 | V8-S3 | 2.234 | 2.320 |
| V3-S4 | 2.589 | 2.565 | V8-S10 | 2.591 | 2.244 |
| V3-S5 | 2.478 | 2.476 | V8-S11 | 2.477 | 2.251 |
| V4-S2 | 2.504 | 2.515 | V9-S9 | 2.504 | 2.444 |
| V4-S4 | 2.513 | 2.502 | V9-S10 | 2.513 | 2.461 |
| V4-S6 | 2.270 | 2.282 | V9-S12 | 2.271 | 2.296 |
| V4-S7 | 2.320 | 2.313 | V9-S13 | 2.320 | 2.300 |
| V5-S6 | 2.317 | 2.309 | V10-S12 | 2.318 | 2.316 |
| V5-S7 | 2.271 | 2.277 | V10-S13 | 2.272 | 2.276 |

**Table S4.** Comparison of HER activity for *V_S_*-VS_2_-VS superlattices with other reported catalysts in 0.5 M H_2_SO_4_.

| **Catalyst** | **Overpotential at 10 mA/cm^2^ (mV)** | **Tafel**  **Slope**  **(mV/dec)** | **Reference** |
| --- | --- | --- | --- |
| *V_S_*-VS_2_-VS SLs | 46 | 36.9 | *This work* |
| Mo-Co_9_S_8_@C | 98 | 34.6 | *Adv. Energy Mater.* ***2020****, 10, 1903137.* |
| 2H-MoS_2_-2.5 M KCl | 158.8 | 54.3 | *Adv. Mater.* ***2024****, 36, 2304808.* |
| FeP NFSLs | 71 | 61 | *Adv. Mater.* ***2022****, 34, 2109145.* |
| FD-MoS_2_ | 164 | 36 | *Nat. Commun.* ***2022****, 13, 2193.* |
| WS_2_/WO_x_S_y_/WO_3_ | 72 | 42 | *Adv. Mater.* ***2024****, 2314031.* |
| interlayer-confined NiFe@MoS_2_ | 67 | 26.8 | *Adv. Mater.* ***2023****, 35, 32.* |
| 1T-VS_2_ | 68 | 34 | *Adv. Mater.* ***2015****, 27, 5605 – 5609.* |
| RuO_2_-WC NPs | 58 | 66 | *Angew. Chem. Int. Ed.* ***2022****, 61, 21* |
| WS_2_@graphene | 117 | 56 | *Adv. Mater.* ***2020****, 32, 2002584.* |
| (N, PO_4_^3-^)-MoS_2_/VG | 85 | 42 | *Angew. Chem. Int. Ed.* ***2019****,*  *131, 16435 – 16442* |
| Zn_0.30_Co_2.70_S_4_ | 80 | 47.5 | *J. Am. Chem. Soc.* ***2016****, 138, 1359.* |
| WS_2_ MSLs | 60 | 40 | *Nat. Commun.* ***2017****, 12, 5070.* |
| WS_2_ nanodots | 209 | 63 | *Adv. Mater.* ***2018****, 30, 1705509.* |
| SV-MoS_2_ | 170 | 60 | *Nat. Mater.* ***2016****, 15, 48.* |
| 1T′-MoS_2_ | 175 | 100 | *Nat. Chem.* ***2018****, 10, 638.* |
| CoNi@NC | 142 | 105 | *Angew. Chem. Int. Ed.* ***2015****,*  *54, 2100* |
| FeS_2_-rGO | 139 | 66 | *Adv. Energy Mater.* ***2017****, 7, 1700482.* |
| Co–WS_2_ | 240 | 49 | *Energy Environ. Sci.* ***2018****, 11, 2270.* |
| mPF-Co–MoS_2_ | 156 | 74 | *Nat. Commun.* ***2017****, 8, 14430.* |
| V SACs@1T-WS_2_ | 185 | 61 | *Nat. Commun.* ***2021****, 12, 709* |

**Reference**

[1] G. Kresse, J. Hafner, *Physical review B* **1993**, 47, 558.

[2] G. Kresse, J. Furthmüller, *Comput. Mater. Sci* **1996**, 6, 15.

[3] G. Kresse, J. Furthmüller, *Physical review B* **1996**, 54, 11169.

[4] P. E. Blöchl, *Physical review B* **1994**, 50, 17953.

[5] S. Grimme, *J. Comput. Chem.* **2006**, 27, 1787.

[6] S. L. Dudarev, G. A. Botton, S. Y. Savrasov, C. Humphreys, A. P. Sutton, *Physical Review B* **1998**, 57, 1505.

[7] J. Zhou, W. Zhang, Y.-C. Lin, J. Cao, Y. Zhou, W. Jiang, H. Du, B. Tang, J. Shi, B. Jiang, *Nature* **2022**, 609, 46.

[8] J. Zhou, C. Zhu, Y. Zhou, J. Dong, P. Li, Z. Zhang, Z. Wang, Y.-C. Lin, J. Shi, R. Zhang, *Nat. Mater.* **2023**, 22, 450.
